# Geographic disparities in trends of thyroid cancer incidence and mortality from 1990 to 2019 and a projection to 2030 across income-classified countries and territories

**DOI:** 10.7189/jogh.13.04108

**Published:** 2023-09-29

**Authors:** Chenran Wang, Zheng Wu, Lin Lei, Xuesi Dong, Wei Cao, Zilin Luo, Yadi Zheng, Fei Wang, Yongjie Xu, Liang Zhao, Jufang Shi, Jiansong Ren, Jibin Li, Yawei Zhang, Wanqing Chen, Ni Li

**Affiliations:** 1 Office of Cancer Screening, National Cancer Center/National Clinical Research Center for Cancer/Cancer Hospital, Chinese Academy of Medical Sciences and Peking Union Medical College, Beijing, China; 2 Department of Cancer Prevention and Control, Shenzhen Center for Chronic Disease Control, Shenzhen, China; 3 Department of Cancer Prevention and Control, National Cancer Center/National Clinical Research Center for Cancer/Cancer Hospital, Chinese Academy of Medical Sciences and Peking Union Medical College, Beijing, China; 4 Collaborative Innovation Center for Cancer Personalized Medicine, Nanjing Medical University, Nanjing, China

## Abstract

**Background:**

The rising incidence of thyroid cancer (TC) has generated growing concern globally; yet there are no studies examining whether this incidence was followed by a rise in related mortality. We aimed to comprehensively quantify current trends and future projections of TC incidence and mortality, and to explore the association between the TC burden and socioeconomic inequality in different income strata.

**Methods:**

We obtained incidence and mortality data on TC and population from the 2019 Global Burden of Disease (GBD) study and the United Nations’ World Population Prospects 2022. We applied an age-period-cohort (APC) model to estimate the overall annual percentage change (net drift) and age, period, and cohort effects from 1990 to 2019, and also constructed a Bayesian APC model to predict the TC burden through 2030.

**Results:**

Over a third of global TC cases belonged to the high-income group. From 1990 to 2019, net drifts of TC incidence were >0 in all income groups, while a modest reduction (net drift <0) in mortality was observed in most income groups, except for the lower-middle-income group. Unfavourable age, period, and cohort effects were most notable in Vietnam, China, and Korea. The age-standardised incidence rate (ASIR) is predicted to increase whereas the age-standardized mortality rate (ASMR) is expected to decrease globally between 2020 and 2030, with geographic heterogeneity being detected across income groups. We observed a positive correlation between ASIR and universal health coverage index and health worker density, but a negative one between ASMR and the two indicators, primarily in upper-middle-income and high-income countries.

**Conclusions:**

Opposite patterns in incidence and mortality of TC raise concerns about overdiagnosis, particularly in upper-middle-income and high-income countries. Discrepancies in the distribution of health service accessibility, including diagnostic techniques and therapeutic care, should be addressed by narrowing health inequalities in the TC burden across countries.

Over the last three decades, the incidence rate of thyroid cancer (TC) increased by 87.5% globally, generating significant concern [1]. 2-fold rates were observed among females compared to males, and substantial variability in TC incidence and mortality also existed across countries with varying income levels; in 2019, 34.8% of newly diagnosed TC cases globally occurred in high-income countries, but an estimated 36% occurred in lower-middle-income countries [1].

These large cross-country disparities might be related to differences in national screening and diagnostic techniques, access to health care, environmental risk factors, and individual lifestyles [2]. Notably, concerns have been raised that over-screening and over-diagnosis may contribute significantly to an increased incidence of TC, especially in high-income countries with adequate medical practices and robust health service capabilities. Evidence from high-income countries such as South Korea [3], Japan [4], and the USA [5] suggest over-diagnosis may be associated with an elevated TC risk, where the progress in diagnostic imaging, thyroid surgery, and national screening programs increased the false-positive rates of TC. However, other studies suggest that other risk factors such as obesity [6], radiation exposure [7], and iodine supply [8] could also be potential aetiologies. Whether TC incidence and related morality truly increased in different income-level countries remains to be determined.

A quantification of levels and trends in TC incidence and mortality in different income strata can provide important insights into whether the TC burden and socioeconomic inequality are ecologically correlated. Some studies have examined the variation trends in TC incidence, mortality, and disability-adjusted life years on a global scale [9-11]. However, there are few studies on low- and lower-middle-income countries specifically, and none that assessed socioeconomic disparities across countries. Furthermore, few analyses have differentiated the relative contributions of age, period, and cohort effects on TC incidence and mortality. Since the occurrence and development of TC could be relevant not only to age-related conditions (age effects), but also to technological advances in early screening and diagnosis (period effects) [12], and risk factor exposures (cohort effects) in utero or early life [13], the potential association of these effects with TC incidence and mortality trends across countries should be explored. Additionally, evidence-informed projections of the TC burden are key in planning health resources allocation for TC primary prevention, screening, and early diagnosis [14]. Nevertheless, studies have not yet assessed the impact of current TC trends will have on future projections.

To address these knowledge gaps, we aimed to explore the contributions of age, period, and cohort effects of TC incidence and mortality trends in different income groups, and to predict the future TC burden through 2030 based on data from the most up-to-date 2019 Global Burden of Disease (GBD) study. We further explored the association between the accessibility of health service and TC incidence and mortality rates in each income stratum.

## METHODS

### Type of study

We conducted a secondary analysis of GBD 2019 data, the methods of which we have elaborated elsewhere [1]. The waiver of informed consent of the GBD study has been reviewed and approved by the University of Washington Institutional Review Board. We followed the Guidelines for Accurate and Transparent Health Estimates Reporting (GATHER) statement in reporting this study (Checklist S1 in the **Online Supplementary Document**).

### Data sources

#### TC incidence and mortality, and population data

We extracted annual incidence and mortality data of TC, and population counts from 1990 to 2019 by sex and age group (20 age groups, from 0-4 years to >95 years) in the globe, by four income groups (low-income, lower-middle-income, upper-middle-income, and high-income groups, as categorised by the World Bank) and 201 income-classified countries and territories (based the Global Health Data Exchange [15]) (Table S1 in the **Online Supplementary Document**). We also obtained corresponding 95% uncertainty intervals (UI) calculated in posterior simulation of 1000 drawings to estimate uncertain distributions. The diagnosis codes of TC were C73-C73.9, D09.3, D09.8, D34-D34.9, D44.0, Z85.850, 193-193.9, and 226-226.9 as per the International Classification of Diseases (ICD) mapped to the GBD 2019 cause list [1,16]. To further forecast the incidence and mortality burden of TC, we retrieved the projected population data between 2020 and 2030 by age group for the four World Bank-classified income groups from the United Nations’ World Population Prospects 2022 [17] and for the 201 income-classified countries and territories from the GBD 2019 population estimates, respectively (the age-stratified projected population data in the World Bank income groups are not available to the GBD 2019).

#### Universal health coverage

We assessed the accessibility of health services using the universal health coverage (UHC) index and health workforce density at the national/regional level in 201 countries and territories.

UHC refers to high-quality health services and freedom from financial hardship being accessible to all [18]. To monitor the effective coverage of health systems at the population level across settings, the GBD collaborators developed a nationally and regionally comparable measurement framework incorporating 23 effective health coverage indicators. These indicators were categorised into five health service domains: promotion, prevention, treatment, rehabilitation, and palliation. The UHC index was measured on a scale of 1 to 100, with higher scores indicating better health services coverage. The values for 201 countries and territories in 2019 are provided in Table S2 in the **Online Supplementary Document**.

#### Health workforce density

Human resource for health (HRH) is associated with sociodemographic development and population-level health outcomes [19]. The GBD 2019 study used health workforce density (workers per 10 000 population) as the indicator to assess the level of HRH across countries and territories, estimated based on employment status and current occupation data from World Health Organization’s Global Health Observatory, cross-sectional surveys, and censuses [20]. The values of health workforce density ranged from 14 (Somalia, low-income country) to 696 (Sweden, high-income country) workers per 10 000 population among 201 countries and territories (Table S2 in the **Online Supplementary Document**).

#### Selected exemplary countries

To better characterise the current trends and future projections of TC incidence and mortality in specific countries, we selected two to three exemplary countries from each income stratum. For upper-middle-income and high-income countries, selected countries needed to fulfil that they had high coverage of national or regional cancer registration and vital statistics, and evidence showed that the epidemiology of TC was of special concern at the national level [20]. Since high-quality national cancer registry data were less available to some low-income and lower-middle-income countries, we calculated the estimated annual percentage change (EAPC) of age-standardised incidence rate (ASIR) as an auxiliary indicator, with those with the high EAPC being considered alternative countries (Text S1, Figures S1-S4 in the **Online Supplementary Document**). The enrolled ten exemplary countries included two low-income countries (Nepal and Uganda), two lower-middle-income countries (Vietnam and India), three upper-middle-income countries (China, Ecuador, and Turkey), and three high-income countries (the Republic of Korea, Australia, and the USA).

### Statistical analyses

#### Age-period-cohort analyses

We used the parametric statistical age-period-cohort (APC) model to explore the time trends in TC incidence and mortality produced by age, period, and cohort effects from 1990 to 2019. In the classic APC model, TC counts were assumed to follow a Poisson distribution, and the log-liner Poisson model was fitted with log (λij) = μ + αi + βj + γk [21], where λij represented the TC incidence or mortality rate, μ the mean effect, and αi, βj, and γk the age, period, and cohort effects, respectively. Net drift, the most the APC model’s most important parameter, can be expressed as the estimated annual percentage change of TC incidence and mortality, reflecting the overall log-liner temporal trend by period and cohort over the study period. The value of zero represents the fitted temporal trend is stable over time. Local drift represents the estimated annual percentage change specific to age group, and it can be interpreted as the consequence of trends in cohort effects. Longitudinal age curves that reflect the age-related natural history are fitted to represent the age effects, and the longitudinal age-specific rates in the reference cohort are adjusted for period deviations. The period/cohort effects are represented by risk ratio (RR), which are calculated as the ratio of age-specific rates in each period/cohort relative to the reference period/cohort [22].

For the APC analyses, we arranged data on TC incidence, mortality, and population counts into six consecutive five-year periods (ie, 1990 to 1994, 1995 to 1999, 2000 to 2004, 2005 to 2009, 2010 to 2014, and 2015 to 2019) from 1990 to 2019, with the 2000-2004 survey year being defined as the reference period, and into 14 successive five-year age intervals from 20-24 years to 85-89 years, with those aged 50-54 years being set as the reference age group. We calculated the average incidence, death, and population counts in each five-year period to estimate rates of incidence and mortality over the specific period. The input data consisted of 19 (C birth cohorts = A age groups + P periods-1) partially overlapping birth cohorts from 1901-1909 (median: the 1905 cohort) to 1991-1999 (median: the 1995 cohort), with those born from 1946 to 1954 (median: the 1950 cohort) being identified as the reference birth cohort (see for Lexis diagram of APC model in Table S3 in the **Online Supplementary Document**). We estimated age, period, and cohort parameters of APC model by the age-period-cohort Web Tool of the National Cancer Institute [22] and tested the significance of the estimable parameters and functions with Wald’s χ^2^ test.

#### Bayesian age-period-cohort model

We projected absolute number, rates, and age-standardised rates for TC incidence and mortality from 2020 to 2030 following the well-established Bayesian APC model [23,24]. In the Bayesian model, unknown parameters are treated random variables with prior distributions, and the observed data from available sample are combined with prior knowledge to estimate the posterior distribution. Considering that the effects might be similar between adjacent time bands, second-order random walk prior based on the assumption of a liner time trend in period effects is used to adjust for the excessive dispersion [24]. We arranged data on TC incidence and mortality into 30 consecutive one-year periods from 1990 to 2019, and 20 successive five-year age intervals from 0-4 years to >95 years; we then collated the corresponding population data into 41 consecutive one-year periods from 1990 to 2030 (see data sources). We constructed the Bayesian APC analysis with integrated nested Laplace approximation. We performed the statistical analysis using the package BAPC (version 0.0.34) in R, version 4.2.1 (R Core Team, Auckland, New Zealand).

#### Correlation and regression analyses

We evaluated the correlations of TC ASIR and age-standardised mortality rates (ASMR) with UHC index and HCH indicator (all health workers density) in 201 income-stratified countries and territories by Pearson correlation analysis. We further sequentially fitted a simple linear regression with ASIR and ASMR on the y-axis and UHC index and all health workers density on the x-axis to detect the inter-dependency between the TC burden and national/regional health services accessibility. We calculated coefficients of correlation (r) and regression (β) as statistics of potential association.

We tested the distribution of continuous variables for normality using the Shapiro-Wilk test. In the sensitivity analysis, we selected sociodemographic index (SDI) developed by GBD 2019 as an alternative indicator of income level to test the robustness of the association between health service accessibility and the TC burden [25]. We performed data collation and statistical analyses were performed with SAS 9.4 (SAS Institute Inc., Cary, North Carolina, USA). The statistical tests were two-sided and the significance level of α was 0.05.

## RESULTS

### Relative change in TC incidence and mortality, 1990-2019

#### By income group

Globally, the number of newly-diagnosed TC cases increased by 167% from 1990 (n = 87 583) to 2019 (n = 233 847), and the number of TC deaths increased by 98.4% period (2019: n = 45 576). Compared with males, females had 2.7 and 2.1 folds higher incidence rate in 1990 and 2019, respectively. Over a third of global TC cases occurred in World Bank-classified high-income countries (48.4% in 1990 and 34.8% in 2019), while lower-middle-income countries had the largest proportion (36%) of global deaths in 2019. From 1990 to 2019, the high-income group had the highest rates of incidence and mortality, followed by the upper-middle-income group, while the lower-middle-income group had the lowest mortality in 1990 (0.3 per 100,000 person-years), but was replaced by the low-income group in 2019 (0.4 per 100,000 person-years) (**Table 1**).

**Table 1:** Trends in incidence and mortality of thyroid cancer across income-classified regions and countries, 1990-2019

	1990				2019				1990-2019		
	**n (95% UI)**	**Per cent of the globe**	**Rate per 100 000 (95% UI)**	**Female/male ratio of rate**	**n (95% UI)**	**Per cent of the globe**	**Rate per 100 000 (95% UI)**	**Female/male ratio of rate**	**Per cent change of number**	**Per cent change of rate**	**Net drift from APC model, per cent per year (95% UI)**
**Incidence**											
Global	87 583 (82236, 92717)	100.0	1.6 (1.5, 1.7)	2.7	233 847 (211 637, 252 807)	100.0	3.0 (2.7, 3.3)	2.1	167.0	87.5	1.24 (1.19, 1.29)
**WB income level**											
Low-income	3127 (2243, 4200)	3.6	0.9 (0.7, 1.3)	3.8	7640 (6068, 9332)	3.3	1.1 (0.9, 1.3)	3.4	144.4	22.2	0.3 (0.14, 0.47)
Lower-middle-income	17 477 (15 042, 19 573)	20.0	0.9 (0.8, 1.0)	3.5	63914 (52 812, 71 049)	27.3	2.0 (1.7, 2.2)	3.0	265.7	122.2	1.93 (1.87, 1.99)
Upper-middle -income	24 584 (22 549, 26 813)	28.1	1.2 (1.1, 1.3)	3.0	80 749 (72 019, 92 017)	34.5	3.1 (2.7, 3.5)	1.9	228.5	158.3	1.98 (1.87, 2.08)
High-income	42 347 (40 898, 43 453)	48.4	4.2 (4.1, 4.4)	2.1	81 412 (74 003, 89 158)	34.8	6.8 (6.2, 7.4)	1.7	92.3	61.9	0.94 (0.83, 1.05)
**10 income-classified exemplar countries**											
Nepal	129 (90 184)	0.1	0.7 (0.5, 0.9)	3.7	490 (341, 681)	0.2	1.6 (1.1, 2.2)	3.4	278.8	128.6	2.12 (1.36, 2.88)
Uganda	70 (50, 98)	0.1	0.4 (0.3, 0.6)	3.0	444 (291, 627)	0.2	1.1 (0.7, 1.5)	2.7	532.7	175.0	3.27 (2.33, 4.22)
Vietnam	1102 (808, 1469)	1.3	1.6 (1.2, 2.2)	3.6	7789 (5009, 10487)	3.3	8.1 (5.2, 10.9)	2.6	606.7	406.3	4.86 (4.62, 5.1)
India	5989 (4919, 7803)	6.8	0.7 (0.6, 0.9)	3.7	23 823 (19 467, 28 645)	10.2	1.7 (1.4, 2.1)	2.9	297.8	142.9	2.16 (2.05, 2.28)
China	10 030 (8399, 11 907)	11.5	0.8 (0.7, 1.0)	3.3	39 079 (32 279, 47 658)	16.7	2.7 (2.3, 3.4)	1.5	289.6	237.5	2.74 (2.52, 2.96)
Ecuador	96 (81, 152)	0.1	1.0 (0.8, 1.5)	3.8	739 (551, 988)	0.3	4.2 (3.1, 5.6)	3.6	671.2	320.0	4.11 (3.44, 4.79)
Turkey	892 (651, 1138)	1.0	1.5 (1.1, 1.9)	2.8	3271 (2493, 4323)	1.4	4.0 (3.1, 5.3)	2.0	266.5	166.7	1.92 (1.66, 2.19)
Korea	693 (556, 1481)	0.8	1.6 (1.3, 3.3)	3.4	5209 (3565, 6623)	2.2	9.8 (6.7, 12.4)	3.0	651.2	512.5	5.43 (4.74, 6.12)
Australia	462 (422, 509)	0.5	2.7 (2.5, 3)	2.1	1580 (1204, 2073)	0.7	6.4 (4.9, 8.4)	1.9	241.8	137.0	2.78 (2.43, 3.13)
USA	11 690 (11 268, 12 063)	13.3	4.6 (4.4, 4.8)	1.5	26 270 (22 444, 30 610)	11.2	8.0 (6.8, 9.3)	1.2	124.7	73.9	1.13 (1.05, 1.21)
**Death**											
Global	22 966 (21 554, 25 228)	100.0	0.4 (0.4, 0.5)	2.0	45 576 (41 290, 48 775)	100.0	0.6 (0.5, 0.6)	1.4	98.4	50.0	-0.27 (-0.33, -0.20)
**WB income level**											
Low-income	1375 (1068, 1791)	6.0	0.4 (0.3, 0.5)	3.0	2554 (2050, 3057)	5.6	0.4 (0.3, 0.4)	2.5	85.7	0.0	-0.75 (-0.98, -0.51)
Lower-middle-income	6459 (5779, 7498)	28.1	0.3 (0.3, 0.4)	2.5	16 422 (14 284, 18 108)	36.0	0.5 (0.4, 0.6)	1.8	154.3	66.7	0.35 (0.24, 0.45)
Upper-middle -income	7415 (6889, 8431)	32.3	0.4 (0.3, 0.4)	2.5	15 192 (13 569, 16 788)	33.3	0.6 (0.5, 0.6)	1.2	104.9	50.0	-0.7 (-0.82, -0.58)
High-income	7703 (7218, 7950)	33.5	0.8 (0.7, 0.8)	2.0	11 379 (9832, 12 142)	25.0	0.9 (0.8, 1)	1.4	47.7	12.5	-0.98 (-1.17, -0.78)
**10 income-classified exemplar countries**											
Nepal	57 (43, 76)	0.2	0.3 (0.2, 0.4)	2.0	155 (117, 207)	0.3	0.5 (0.4, 0.7)	1.5	172.5	66.7	0.53 (-0.59, 1.66)
Uganda	33 (25, 45)	0.1	0.2 (0.1, 0.3)	2.0	139 (98 176)	0.3	0.3 (0.2, 0.4)	1.3	321.3	50.0	2.28 (0.95, 3.63)
Vietnam	373 (279, 602)	1.6	0.5 (0.4, 0.9)	2.7	1204 (925 1533)	2.6	1.2 (1, 1.6)	1.5	222.6	140.0	2.15 (1.68, 2.62)
India	2461 (2068, 3250)	10.7	0.3 (0.2, 0.4)	2.0	7075 (5979, 8315)	15.5	0.5 (0.4, 0.6)	1.5	187.5	66.7	0.51 (0.34, 0.68)
China	3319 (2862, 4133)	14.5	0.3 (0.2, 0.3)	2.0	7239 (6012, 8476)	15.9	0.5 (0.4, 0.6)	0.7	118.1	66.7	-0.63 (-0.85, -0.42)
Ecuador	42 (36, 67)	0.2	0.4 (0.4, 0.7)	3.0	217 (155, 282)	0.5	1.2 (0.9, 1.6)	2.6	414.7	200.0	1.61 (0.29, 2.95)
Turkey	257 (196, 316)	1.1	0.4 (0.3, 0.5)	2.0	455 (353, 636)	1.0	0.6 (0.4, 0.8)	1.2	77.1	50.0	-1.96 (-2.68, -1.23)
Korea	140 (115, 313)	0.6	0.3 (0.3, 0.7)	2.0	580 (426, 679)	1.3	1.1 (0.8, 1.3)	2.1	314.3	266.7	1.37 (0.53, 2.22)
Australia	79 (73, 85)	0.3	0.5 (0.4, 0.5)	1.5	187 (157, 205)	0.4	0.8 (0.6, 0.8)	1.1	136.6	60.0	0.6 (-0.98, 2.21)
USA	1252 (1167, 1301)	5.5	0.5 (0.5, 0.5)	1.5	2466 (2231, 2582)	5.4	0.8 (0.7, 0.8)	1.1	96.9	60.0	0.20 (-0.23, 0.63)

WB – World Bank, APC – age-period-cohort, UI – uncertainty interval, CI – confidence interval

#### By income-classified exemplary country

**Figure 1:**
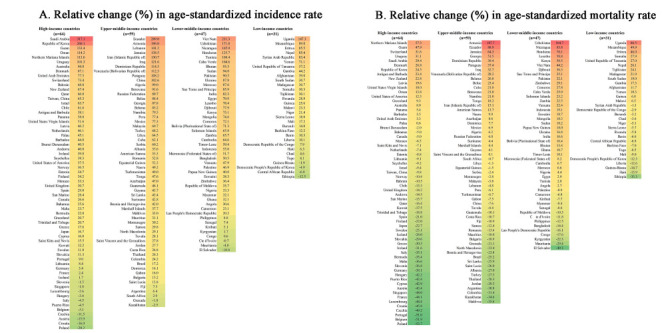
Relative change in age-standardized incidence and mortality rates of thyroid cancer in 201 income-classified countries and territories from 1990 to 2019. **Panel A.** Relative change (%) in age-standardized incidence rate. **Panel B.** Relative change (%) in age-standardized mortality rate.

Among 201 income-classified countries, 27 (87.1%) low-income, 44 (93.6%) lower-middle-income, 57 (96.7%) upper-middle-income, and 53 (82.8%) high-income countries showed an increasing trend of ASIR between 1990 and 2019, while 17 (54.8%), 14 (29.8%), 33 (55.9%), and 45 (70.3%) countries in each income stratum showed a reduction in ASMR. We observed an opposite temporal trend of increasing ASIR, but simultaneously decreasing ASMR in 13 (41.2%) low-income, 11 (23.4%) lower-middle-income, 31 (52.5%) upper-middle-income, and 34 (53.1%) high-income countries (**Figure 1**). We found simultaneous increases in TC incidence and mortality rates from 1990 to 2019 in the ten exemplary countries, with those in Korea (512.5% for incidence rate and 266.7% for mortality rate) being the most intense; we found an opposite trend in ASIR and ASMR in two upper-middle-income countries, China and Turkey (**Table 1**, **Figure 1**).

### Net drift and local drift of TC incidence and mortality, 1990-2019

#### By income group

**Figure 2:**
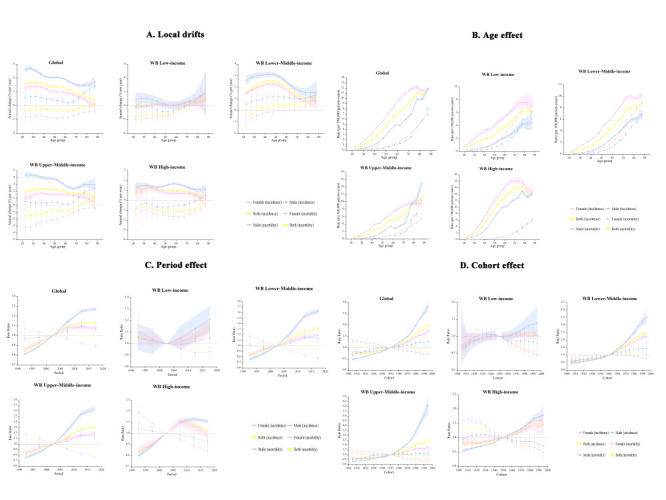
Local drifts, and age, period, and cohort effects on thyroid cancer incidence and mortality by income level. **Panel A.** Local drifts of thyroid cancer incidence and mortality by income level. **Panel B.** Age effect of thyroid cancer incidence and mortality by income level. **Panel C.** Period effect of thyroid cancer incidence and mortality by income level. **Panel D.** Cohort effect of thyroid cancer incidence and mortality by income level.

Globally, the APC model estimated a net drift of TC incidence at 1.24% (95% confidence interval (CI) = 1.19, 1.29) per year from 1990 to 2019, ranging from 0.30% (95% CI = 0.14, 0.47) in the low-income group to 1.98% (95% CI = 1.87, 2.08) in the upper-middle-income group; the local drifts of incidence were predominantly above zero in most age groups for both sexes at the global level and in all four income groups (except females aged 20-30 years in the low-income group). The annual increase in incidence rate among males exceeded females across all age groups, regardless of income level (**Table 1**, **Figure 2**, panel A).

There was an overall slight annual decrease (net drift = -0.27%; 95% CI = -0.33, -0.20) in TC mortality rate at the global level, while the trend was reversed (0.35%; 95% CI = 0.24,0.45) in the lower-middle-income group (**Table 1**). The difference test between the net drift and local drift of mortality was statistically significant (*P* < 0.05) at the global level and in all four income groups **(**Table S4 in the **Online Supplementary Document****)**. We observed a decreasing trend of mortality was observed in the whole population across all age groups among the high-income group. The local drift of mortality showed wide disparities between sexes in the upper-middle-income group of countries: the decreasing tendency of mortality rate progressively attenuated with age in females, while the increasing trend intensified in males aged >60 years (**Figure 2**, panel A).

#### By income-classified exemplary country

We observed increasing net drifts of TC incidence in all exemplary countries, with a similar overall net drift between China (2.74%; 95% CI = 2.52, 2.96) and Australia (2.78%; 95% CI = 2.43, 3.13). Turkey (-1.96%; 95% CI = -2.68, -1.23) and China (-0.63%; 95% CI = -0.85, -0.42) had annual percentage decreases in mortality between 1990 and 2019, while the net drifts in mortality were essentially flat in Nepal, Australia, the USA (**Table 1**). The difference test between the net drift and local drift was statistically significant for incidence in Vietnam, India, Korea, and the USA, and for mortality in India and China (*P* < 0.05) (Table S5 in the **Online Supplementary Document****)**.

Consequently, the inconsistent direction of mortality change between the net drift and overall relative change, and the statistically significant difference between the net and local drifts both suggested that the conventional metrics such as percentage change or EAPC, could not fully interpret the temporal trends of TC incidence and mortality across age groups. Thus, the identification and differentiation of age, period, and cohort effects were required.

### Age, period, and cohort effects on TC incidence and mortality, 1990-2019

#### Age effect

Age effects on TC incidence continuously increased with age among the whole population in the lower-middle-income and upper-middle-income groups, while the incidence rate peaked in adults aged 75-79 years and decreased in the later years of life in the high-income group. The overall incidence risk increased with income level, with high-income countries showing higher incidence across all age groups compared with other income groups. Except for upper-middle-income countries, the incidence rate was consistently higher in females than males across all age groups in the globe and other three income groups. We found similar patterns of age effects on TC mortality globally, in all four income groups, and in ten exemplary countries, with increasing mortality risk with age in both sexes (**Figure 2**, panel B and Figure S5A-D in the **Online Supplementary Document**).

#### Period effect

Period effect showed increasing risk in TC incidence for both sexes in the globe and the middle-income groups from 1990 to 2019. We saw no obvious period effect in the low-income group over the 1990-2000 period (95% CI of RR contains 1.0), but found increasing period risks in both sexes over the past decade. The high-income group had an initial increased period risk of incidence in 1990-2010, but remained constant in males and experienced reduction in females after 2005. Decreasing period risk of TC mortality occurred in the whole population at the global level and in all four income groups in the past three decades (**Figure 2**, panel C).

We observed increases in period risks of TC incidence and mortality in Vietnam between 1990 and 2019, but an increasing incidence risk and a reversed, declining mortality trend in Turkey. China presented typical characteristics of upper-middle-income countries in terms of period effects, with worsening period risks in incidence in both sexes and inverse trends in mortality between males and females from 1990 to 2019. Korea stood out as a high-income country with increasing risks of incidence and mortality before the period 2005-2009, but persistently steep declining period risks post-2009. Australia had gradual increases in RRs of incidence and mortality from 1990 to 2014, with decreased period effects in the latest five years (Figure S5, panels A-D in the **Online Supplementary Document**).

#### Cohort effect

Cohort effects showed similar trends as period effects. There were overall increasing cohort risks of incidence globally and in the middle-income and high-income groups. Compared with individuals born in the referent 1950 cohort, the cohort RR of incidence for those born in the 1995 cohort ranged from 1.0 (95% CI = 0.9, 1.2) in the low-income group to 2.6 (95% CI = 2.2, 2.9) in the upper-middle-income group. We found declining cohort risks of mortality in individuals born after 1930 globally, and in those born from 1935, 1975, 1930, and 1910 on in the low-income, lower-middle, upper-middle, and high-income groups, respectively (**Figure 2**, panel D).

The cohort effects of incidence showed a single-peaked curve in Vietnam, Korea, and Australia. However, we observed a notable decreasing risk of mortality in successive birth cohorts in China (from 1935) and Turkey (from 1925) (Figure S5A-D in the **Online Supplementary Document**).

### Projections of TC incidence and mortality up to 2030

#### By income group

**Figure 3:**
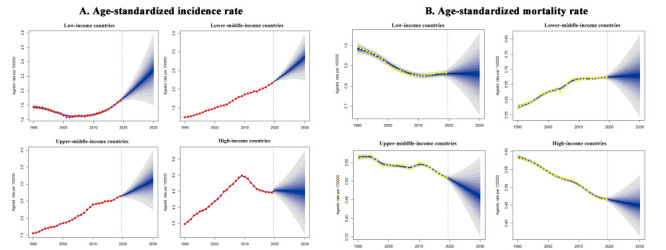
The temporal trends of age-standardized rates of incidence and mortality for thyroid cancer in four income groups from 1990 to 2030. **Panel A.** The temporal trends of age-standardized incidence rate. **Panel B.** The temporal trends of age-standardized mortality rate.

Globally, the model predicted that TC cases will increase by 44.1% from 2019 to 2030 (n = 337 000), with ASIR per 100 000 increasing by 16.3%, from 2.83 in 2019 to 3.29 in 2030; simultaneously, TC deaths are estimated to increase to 60 000 in 2030, a projected 31.7% increase in absolute numbers, but a 2.7% decrease in ASMR compared with 2019 (Tables S6-8 and Figures S6A-B, S7A, and S8A in the **Online Supplementary Document**). The increase in ASIR between 2020 and 2030 will be most pronounced in upper-middle-income group (27.4%). Conversely, the ASMR is predicted to decrease in the upper-middle-income and high-income groups in the next decade, with the most notable decrease occurring in the high-income group (-6.6%) (**Figure 3**, panels A-B). We projected an increase in TC cases in 55-79-year-old age group in upper-middle-income group and the 45-69-year-old age group in lower-middle-income group (Figure S7B-E in the **Online Supplementary Document**). The projected age-standardised rates of incidence and mortality by age group globally and across income groups showed heterogeneity (Figures S9-18 in the **Online Supplementary Document**).

#### By income-classified exemplary country

Among the ten exemplary countries, we predicted the ASIR of TC in Nepal, Uganda, India, Turkey, China, and Vietnam, would increase notably between 2020 and 2030, while a pronounced decrease in ASMR will be observed in most of the exemplary countries except Nepal, Uganda, and India (Figure S6A-B in the **Online Supplementary Document**).

#### Different scenarios of changing patterns from 1990 to 2030

The changing patterns of TC ASIR and ASMR from 1990 to 2030 in the four income groups could be categorised into four scenarios, with the year 2019 as the demarcation (**Figure 3**, panels A-B). The four scenarios are:

− The low-income group experienced an initial decrease in ASIR until 2000, but will see a persistent increase by 2030; ASMR showed an initial decrease and will likely remain at a relatively high level;
− The lower-middle-income group has and will continue to experience a persistent increase in ASIR; it experienced an initial increase in ASMR, yet this rate is predicted to remain stable in the next decade;
− The upper-middle-income group has and will continue to experience a persistent increase in ASIR, but a persistent decrease in ASMR is likely to occur from 2020 to 2030;
− The high-income group experienced an initial increase in ASIR until 2009, while it decreased slightly and will likely remain relatively high from 2018 onwards; the ASMR is estimated to decrease persistently from 1990 to 2030.

### Association between TC age-standardised rates and universal health coverage and health worker density

**Figure 4:**
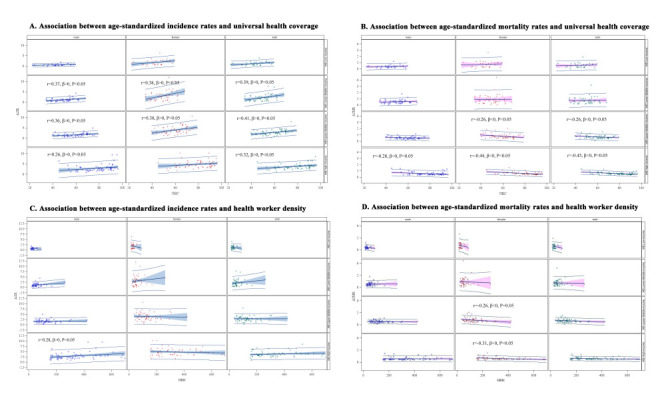
Association between age-standardized incidence and mortality rates of thyroid cancer and universal health coverage and health worker density in 201 income-classified countries in 2019. **Panel A.** Association between age-standardized incidence rates and universal health coverage. **Panel B.** Association between age-standardized mortality rates and universal health coverage. **Panel C.** Association between age-standardized incidence rates and health worker density. **Panel D.** Association between age-standardized mortality rates and health worker density.

We found a significant positive correlation between TC ASIR and UHC index in the low-middle-income, upper-middle-income, and high-income (except for females) groups in 2019 (r >0, *P* < 0.05) (**Figure 4**, panel A), but a significant negative correlation between ASMR and UHC index in the upper-middle (except for males) and high-income groups in 2019 (r <0, *P* < 0.05) (**Figure 4**, panel B). Similarly, we observed the potentially positive association between ASIR and health worker density in males in the high-income group (**Figure 4**, panel C) and opposite negative correlation between ASMR and health worker density among females in the upper-middle-income group and among males in the high-income group (**Figure 4**, panel D).

The associations between age-standardised rates of incidence and mortality for TC and health service accessibility across countries and territories with varying SDI levels remained similar in the sensitivity analysis (Figure S19 in the **Online Supplementary Document**).

## DISCUSSION

We observed an increasing trend in TC incidence and a modest reduction in TC mortality from 1990 to 2019 on a global level, and found wide geographic heterogeneity in temporal variations of TC incidence and mortality across countries with different income levels. We also predicted an increase in ASIR and a decrease in ASMR between 2020 and 2030. We also determined diverging associations between the health services accessibility and ASIR/ASMR in upper-middle-income and high-income countries, indicating large health disparities globally.

Reliance on the overall relative change might be misleading when tracking the temporal trends of TC. Our APC analysis differentiated age, period, and cohort effects, allowing for the improved examination of the TC epidemiological transition. Opposite patterns in global incidence and mortality rates of TC exhibited the typical epidemiological feature of over-diagnosis (ie, the rising incidence rate of TC was not concomitant with the corresponding trend in mortality rate) [26]. From the 1980s onward, the improvement of neck ultrasonography and new imaging technologies led to the growing detection of thyroid millimetric nodules and indolent cancerous lesions, which would not increase mortality risk [12]. The shift of the age-specific curves to an earlier peak was also suggestive of over-diagnosis [26]. We identified similar patterns in age effects, with high-income countries having an overall higher incidence across all age groups compared with other income groups. These important epidemiological characteristics indicated the potential effects of the accessibility of TC-related screening and diagnose techniques, and the elevated prevalence of potential risk factor exposures.

As a high-income country, Korea stood out for its dramatically increasing risks of incidence during 2000-2009 and in cohorts born after the 1980s; however, we found a simultaneous slight increase in mortality, pointing to an expanding role of over-diagnosis. A possible explanation could be that the nationwide cancer screening programme implemented by the Korean government in 1999, which promoted hospitals to offer TC screening with ultrasonography and led to a rapid increase in TC incidence [27]. However, as patients diagnosed with TC could be treated by radical or subtotal thyroidectomy, a relatively slow increase in mortality risk was observed over the same period [27]. Furthermore, since the early-detected TC was predominantly benign or incidental tumour without great clinical significance, subsequent thyroid-replacement therapy and complications such as hypoparathyroidism and vocal-cord paralysis may pose new health risks to patients [28,29]. Notably, we observed favourable period effects in the past five years in Korea, which could be explained by the increased awareness about the impacts of overdiagnosis [30] and in accordance with our prediction analysis. The epidemic of TC in Korea acted as a cautionary tale for other countries to discourage TC screening [27].We did not observe statistically significant net drift of mortality from 1990 to 2019 in other high-income countries such as America and Australia, which suggests that the increased detection of TC was not concomitant with the corresponding rise in mortality rates. Based on evidence on the benefits and harms of screening for TC, the USA Preventive Services Task Force released its statement that TC screening was not recommended in asymptomatic adults [31]. A definition of high-risk populations must be more precisely developed to effectively optimise the TC screening strategies.

China exhibited typical epidemiological features of TC among upper-middle-income countries. The increase in period and cohort risks of TC incidence accelerated in both sexes, while the mortality risk demonstrated an increasing trend in females but inverse decreasing trend among males, indicating over-diagnosis was more common in females in comparison to males. This finding might be partly explained by greater accessibility of health services due to gynaecological and obstetrical requests among females [32]. However, since the diagnosed patients could receive timely treatment, the incidence-based morality risk did not increase correspondingly as expected. Based on population-based registration data, Li et al. [32] reported that substantial geographical variability in TC incidence existed in China. Further correlation analysis showed the important contribution of availability and affordability of health care to the discrepancies of TC over-diagnosis across regions [33], which was highly consistent with our results. Unnecessary thyroid ultrasound included in health examination checklist among urban employers increased the prevalence of subclinical thyroid disease, which may drive the predominant over-diagnosis [34]. Since our Bayesian APC model indicated that China may be further affected by increased TC incidence without appropriate intervention strategies, it is crucial over-diagnosis, including reducing unnecessary TC screening and treatments, is addressed as soon as possible.

Two lower-middle-income countries (Vietnam and India) saw increasing rates of incidence and mortality rates for TC over the past 30 years. The TC burden is expected to continuously increase in India between 2020 and 2030, which might be ascribed to radiation exposure from nuclear weapons testing and nuclear accidents, and iodine intake [35]. Furthermore, there is a lack of quality data on vital statistics and cancer registries in some low-income countries [36], preventing the timely examination of epidemiological transition of TC and evaluation on impact of screening guidelines. For instance, previous studies in Vietnam reported that over-diagnosis accounted for a large proportion of the rising TC incidence and increased the health system burden [37], suggesting a need for the development of population-based regional cancer registries to monitor the evolution of over-diagnosis in low- and middle-income countries with similar demands.

The accessibility of health services is a key determinant of the observed geographic discrepancy in the TC burden across countries [26]. Monitoring the effective coverage of essential health services could help track progress of health gains at national level [38]. Health worker density, proposed by the United Nation’s Sustainable Development Goals, is a critical indicator to measure the recruitment, development, and retention of health workforce, and represents the level of human resources associated with the attainment of UHC [20]. Interestingly, we found a statistically significant association between the ASIR, ASMR and health availability mainly in upper-middle- and high-income countries, but not in low-income ones. This is consistent with previous studies which found that the highest TC incidence rates were commonly observed in areas with accessible thyroid-gland examination practices and predominantly market-oriented health care systems [39,40].

There are causes of geographic variability in TC incidence and mortality [2]. Obesity is widely known modifiable risk factor that may increase the risk of TC, while other studies observed disparities due to sex [41,42]. We also tried to explore the relationship between ASIR and ASMR and the body mass index (BMI) and found it to be associated with an increased mortality risk of TC among females in upper-middle-income countries and among males in high-income countries; however, we did not find such a correlation for low-income and lower-middle-income countries (Figure S20 in the **Online Supplementary Document**). Therefore, the contribution of obesity-associated hormonal changes to the development of TC deserves further exploration. More epidemiological evidence is needed on the effects of potential environmental and lifestyle risk factors, such as radiation exposure [43], iodine supply [8], smoking [44], and red meat consumption [45] on TC risks.

Reasonable predictions can aid policy makers in optimising health resources and assessing the health effects of intervention strategies [14]. Using the Bayesian APC model, we detected geographic heterogeneity of TC incidence and mortality patterns between 1990 and 2030. We found a persistent increase in ASIR, but decrease in ASMR for the 2020-2030 period in upper-middle and high-income groups of countries. This scenario supports the potential effects associated with over-diagnosis, and suggests that the awareness of its substantial affects should be raised in these income groups. Moreover, we observed that the TC burden in low-income countries, including India, Nepal, and Uganda, remained high and is estimated to further increase in the future. The health authorities in these countries should take up a strong leadership role in increasing the quality of health care and improving the accessibility of health service to combat the high TC burden.

### Strength and limitations

We took advantage of the APC parametric framework, where the effects of age, period, and birth cohorts were differentiated and adjusted. The important parameters including net drift, and period and cohort RR helped us explore the TC burden trends and make comparisons of patterns across countries with varying income levels [22]. To predict TC incidence and mortality for the coming decade, we applied a Bayesian APC model (which is useful in making sensible projections [24]) to smooth the age, period, and cohort effects and to prevent the estimated rates from adjacent effects. Compared with the prior studies, we provide a significant contribution with the exploration of the association between the accessibility of health services and TC burden, strengthening the evidence of an urgent need to improve the health resources delivery and to ease the health inequality in lower socioeconomic areas.

Some limitations should be acknowledged. First, we could not reveal distinct epidemiological patterns of TC with different histological types, including papillary, follicular, medullary, and anaplastic TC [11,39]. Second, the lack of complete cancer registries and vital statistics data may lead to wide uncertainty intervals in incidence and mortality estimates in some low- and lower-middle-income countries. The quality of input data would need to be enhanced to improve the research accuracy for these countries. Third, since we assessed the health services accessibility at the national and regional level, we could not avoid ecological bias in the interpretation of the association between health services accessibility and the TC burden.

## CONCLUSIONS

The current trends and future predictions of TC incidence and mortality showed geographic heterogeneity across countries with different income levels. Over-diagnosis may contribute to the increasing incidence of TC globally, especially among upper-middle-income and high-income countries. The discrepancies in health accessibility between high-income and low-income, and lower-middle-income countries should be considered in order to narrow health inequities in the TC burden.

## Additional material


Online Supplementary Document
