# Supplementary material for: Geographic disparities in trends of thyroid cancer incidence and mortality from 1990 to 2019 and a projection to 2030 across income-classified countries and territories
Source: J Glob Health. 2023 Sep 29;13:04108. doi: 10.7189/jogh.13.04108 (PMC10540248; doi:10.7189/jogh.13.04108)
Supplement: Online Supplementary Document [file jogh-13-04108-s001.pdf]

Supporting files for “Geographic disparities in trends of thyroid cancer incidence and mortality from 1990 to 2019 and a projection to 2030 across income-classified countries and territories”

Content

Checklist. Checklist of information that should be included in new reports of global health estimates 3

Supplementary Method. Calculation of estimated annual percentage change........ 5

Table S1. 201 countries and territories classified by the World Bank........ 6

Table S2. Universal health coverage index and human resources value for health in 201 income-stratified countries and territories 7

Table S3. The lexis diagram for the age-period-cohort model........ 12

Table S4. Wald Chi-Square test for local drifts of the age-period-cohort model in four income groups........ 13

Table S5. Wald Chi-Square test for local drifts of the age-period-cohort model in ten exemplary countries........ 14

Table S6. Predicted number (in thousand) of thyroid cancer cases from 1990 to 203016

Table S7. Predicted number (in thousand) of thyroid cancer deaths from 1990 to 203017

Table S8. Age-standardized rates (per 100,000) of incidence and mortality for thyroid cancer in 2019 and 2030 and the percentage change from 2019 to 2030........ 18

Figure S1. The estimated annual percentage change of thyroid cancer age-standardized incidence rate in 31 low-income countries from 1990 to 2019 19

Figure S2. The estimated annual percentage change of thyroid cancer age-standardized incidence rate in 47 lower-middle-income countries from 1990 to 2019........ 20

Figure S3. The estimated annual percentage change of thyroid cancer age-standardized incidence rate in 59 upper-middle-income countries from 1990 to 2019........ 21

Figure S4. The estimated annual percentage change of thyroid cancer age-standardized incidence rate in 64 high-income countries from 1990 to 2019 22

Figure S5. Local drifts, and age, period, and cohort effects on thyroid cancer incidence and mortality in ten income-classified exemplar countries........ 23

Figure S6. The temporal trends in age-standardized incidence and mortality rates of thyroid cancer from 1990 to 2019 and the prediction through 2030 in the globe and ten income-classified exemplar countries 28
Figure S7. The temporal trends in incidence cases of thyroid cancer from 1990 to 2019 and the prediction through 2030 across age groups by income level 30
Figure S8. The temporal trends in death numbers of thyroid cancer from 1990 to 2019 and the prediction through 2030 across age groups by income level 32
Figure S9. The temporal trends of age-standardized incidence rates of thyroid cancer globally by age group from 1990 to 2030 35
Figure S10. The temporal trends of age-standardized incidence rates of thyroid cancer in low-income countries by age group from 1990 to 2030 36
Figure S11. The temporal trends of age-standardized incidence rates of thyroid cancer in lower-middle-income countries by age group from 1990 to 2030 37
Figure S12. The temporal trends of age-standardized incidence rates of thyroid cancer in upper-middle-income countries by age group from 1990 to 2030 38
Figure S13. The temporal trends of age-standardized incidence rates of thyroid cancer in high-income countries by age group from 1990 to 2030 39
Figure S14. The temporal trends in mortality rates of thyroid cancer globally by age group from 1990 to 2030 40
Figure S15. The temporal trends in mortality rates of thyroid cancer in low-income countries by age group from 1990 to 2030 41
Figure S16. The temporal trends in mortality rates of thyroid cancer in lower-middle-income countries by age group from 1990 to 2030 42
Figure S17. The temporal trends in mortality rates of thyroid cancer in upper-middle-income countries by age group from 1990 to 2030 43
Figure S18. The temporal trends in mortality rates of thyroid cancer in high-income countries by age group from 1990 to 2030 44
Figure S19. Association between age-standardized incidence and mortality rates of thyroid cancer and age-standardized summary exposure value of body mass index in 201 income-classified countries in 2019 45
Fiure S20. Sensitivity analysis for the association between age-standardized incidence and mortality rates of thyroid cancer and universal health coverage in 201 SDI-classified countries in 2019 46

Checklist. Checklist of information that should be included in new reports of global health estimates

Item #	Checklist item	Reported on page #
**Objectives and funding**		
**1**	Define the indicator(s), populations (including age, sex, and geographic entities), and time period(s) for which estimates were made.	2-3
**2**	List the funding sources for the work.	13
**Data Inputs**		
For all data inputs from multiple sources that are synthesized as part of the study:		
**3**	Describe how the data were identified and how the data were accessed.	2-3
**4**	Specify the inclusion and exclusion criteria. Identify all ad-hoc exclusions.	3
**5**	Provide information on all included data sources and their main characteristics. For each data source used, report reference information or contact name/institution, population represented, data collection method, year(s) of data collection, sex and age range, diagnostic criteria or measurement method, and sample size, as relevant.	2-3
**6**	Identify and describe any categories of input data that have potentially important biases (e.g., based on characteristics listed in item 5).	2
For data inputs that contribute to the analysis but were not synthesized as part of the study:		
**7**	Describe and give sources for any other data inputs.	N/A
For all data inputs:		
**8**	Provide all data inputs in a file format from which data can be efficiently extracted (e.g., a spreadsheet rather than a PDF), including all relevant meta-data listed in item 5. For any data inputs that cannot be shared because of ethical or legal reasons, such as third-party ownership, provide a contact name or the name of the institution that retains the right to the data.	2-3
**Data analysis**		
**9**	Provide a conceptual overview of the data analysis method. A diagram may be helpful.	3-4
**10**	Provide a detailed description of all steps of the analysis, including mathematical formulae. This description should cover, as relevant, data cleaning, data pre-processing, data adjustments and weighting of data sources, and mathematical or statistical model(s).	3-4
**11**	Describe how candidate models were evaluated and how the final model(s) were selected.	3-4
**12**	Provide the results of an evaluation of model performance, if done, as well as the results of any relevant sensitivity analysis.	N/A

**13**	Describe methods for calculating uncertainty of the estimates. State which sources of uncertainty were, and were not, accounted for in the uncertainty analysis.	3-4
**14**	State how analytic or statistical source code used to generate estimates can be accessed.	3-4
**Results and Discussion**		
**15**	Provide published estimates in a file format from which data can be efficiently extracted.	4-9
**16**	Report a quantitative measure of the uncertainty of the estimates (e.g. uncertainty intervals).	4-9
**17**	Interpret results in light of existing evidence. If updating a previous set of estimates, describe the reasons for changes in estimates.	9-12
**18**	Discuss limitations of the estimates. Include a discussion of any modelling assumptions or data limitations that affect interpretation of the estimates.	12

This checklist should be used in conjunction with the GATHER statement and Explanation and Elaboration document, found on gather-statement.org.

Supplementary Method. Calculation of estimated annual percentage change

We calculated estimated annual percentage change (EAPC) of age-standardized incidence rate (ASIR) from 1990 to 2019 in 201 countries and territories. A liner regression was fitted with the natural logarithm, ie, $y = \alpha + \beta\chi + \varepsilon$, where y is ln ASIR, x is the calendar year, and ε is the error term. The EAPC was defined as 100×(exp (β)-1). The positive values of EAPC and the lower boundary of 95% confidence interval (*CI*) indicated an upward trend of age-standardized rates over the time interval, while the negative values of EAPC and the upper boundary of 95% *CI* indicated an opposite downward trend.

Table S1. 201 countries and territories classified by the World Bank

World Bank income level	Count	Countries and territories
Low-income	31	Democratic People's Republic of Korea, Tajikistan, Haiti, Syrian Arab Republic, Yemen, Afghanistan, Nepal, Central African Republic, Democratic Republic of the Congo, Burundi, Eritrea, Ethiopia, Madagascar, Malawi, Mozambique, Rwanda, Somalia, United Republic of Tanzania, Uganda, Benin, Burkina Faso, Chad, Gambia, Guinea, Guinea-Bissau, Liberia, Mali, Niger, Sierra Leone, Togo, South Sudan
Lower-middle-income	47	Cambodia, Indonesia, Lao People's Democratic Republic, Myanmar, Philippines, Timor-Leste, Viet Nam, Kiribati, Micronesia (Federated States of), Papua New Guinea, Solomon Islands, Vanuatu, Kyrgyzstan, Mongolia, Uzbekistan, Republic of Moldova, Ukraine, Bolivia (Plurinational State of), El Salvador, Honduras, Nicaragua, Egypt, Morocco, Palestine, Tunisia, Bangladesh, Bhutan, India, Pakistan, Angola, Congo, Comoros, Djibouti, Kenya, Zambia, Lesotho, Eswatini, Zimbabwe, Cameroon, Cabo Verde, Côte d'Ivoire, Ghana, Mauritania, Nigeria, Sao Tome and Principe, Senegal, Sudan
Upper-middle-income	59	China, Malaysia, Maldives, Sri Lanka, Thailand, Fiji, Marshall Islands, Samoa, Tonga, Armenia, Azerbaijan, Georgia, Kazakhstan, Turkmenistan, Albania, Bosnia and Herzegovina, Bulgaria, North Macedonia, Montenegro, Romania, Serbia, Belarus, Russian Federation, Argentina, Belize, Cuba, Dominica, Dominican Republic, Grenada, Guyana, Jamaica, Saint Lucia, Saint Vincent and the Grenadines, Suriname, Ecuador, Peru, Colombia, Costa Rica, Guatemala, Mexico, Venezuela (Bolivarian Republic of), Brazil, Paraguay, Algeria, Iran (Islamic Republic of), Iraq, Jordan, Lebanon, Libya, Turkey, Equatorial Guinea, Gabon, Mauritius, Botswana, Namibia, South Africa, American Samoa, Nauru, Tuvalu
High-income	64	Andorra, Antigua and Barbuda, Australia, Austria, Bahamas, Bahrain, Barbados, Belgium, Bermuda,Brunei Darussalam, Canada, Chile, Croatia, Cyprus, Czechia, Denmark, Estonia, Finland, France, Germany, Greece, Greenland, Guam, Hungary, Iceland, Ireland, Israel, Italy, Japan, Kuwait, Latvia, Lithuania, Luxembourg, Malta, Monaco, Netherlands, New Zealand, Northern Mariana, Islands, Norway, Oman, Palau, Panama, Poland, Portugal, Puerto Rico, Qatar, Republic of Korea, Saint Kitts and Nevis, San Marino, Saudi Arabia, Seychelles, Singapore, Slovakia, Slovenia, Spain, Sweden, Switzerland, Taiwan (Province of China), Trinidad and Tobago, United Arab Emirates, United Kingdom, United States of America, United States Virgin Islands, Uruguay

Table S2. Universal health coverage index and human resources value for health in 201 income-stratified countries and territories

Income level	Location name	Universal health coverage index	Human resources for health*
**Low-income**	Central African Republic	22.300	28.769
	Somalia	23.940	13.880
	Chad	31.372	16.422
	Guinea	32.335	13.914
	Niger	35.026	15.928
	Guinea-Bissau	35.707	27.522
	Haiti	35.812	30.721
	Afghanistan	39.295	15.086
	Madagascar	39.691	34.442
	Mali	40.661	21.438
	South Sudan	41.694	23.129
	Burkina Faso	41.797	20.548
	Sierra Leone	42.120	31.345
	Eritrea	42.275	25.612
	Togo	42.809	34.615
	Mozambique	44.044	23.126
	Benin	44.624	25.661
	Democratic Republic of the Congo	45.169	66.374
	Ethiopia	46.522	18.187
	Nepal	47.280	58.667
	Liberia	47.600	40.975
	Tajikistan	47.878	87.256
	Gambia	48.065	29.587
	Yemen	49.046	18.365
	Burundi	49.940	23.588
	Uganda	52.748	30.517
	Democratic People's Republic of Korea	52.838	30.154
	United Republic of Tanzania	55.249	51.726
	Malawi	55.521	34.407
	Syrian Arab Republic	57.565	36.527
	Rwanda	59.359	28.911
**Lower-middle-income**	Vanuatu	34.084	36.320
	Micronesia (Federated States of)	34.474	51.694
	Kiribati	35.736	52.448
	Papua New Guinea	37.767	24.242

	Nigeria	38.339	36.329
	Lesotho	38.737	46.561
	Angola	39.158	48.404
	Pakistan	39.168	43.162
	Solomon Islands	39.333	42.028
	Uzbekistan	42.185	165.657
	Cameroon	42.290	70.639
	Cte d'Ivoire	43.040	33.937
	Lao People's Democratic Republic	43.855	51.654
	Congo	43.904	84.431
	Djibouti	45.287	26.196
	Timor-Leste	45.954	26.978
	India	46.826	45.181
	Myanmar	46.954	58.405
	Mongolia	47.907	127.941
	Comoros	48.139	45.139
	Indonesia	48.728	86.044
	Ghana	49.139	52.765
	Senegal	49.610	22.574
	Bhutan	51.301	91.124
	Kenya	51.647	54.793
	Sudan	51.835	31.539
	Bolivia (Plurinational State of)	52.399	112.451
	Zambia	52.694	48.071
	Kyrgyzstan	52.952	90.581
	Mauritania	53.278	29.778
	Eswatini	53.397	54.436
	Bangladesh	53.883	43.901
	Honduras	54.284	48.311
	Zimbabwe	54.461	48.076
	Philippines	54.712	65.518
	Sao Tome and Principe	54.755	31.885
	Egypt	54.797	65.756
	Ukraine	56.752	261.358
	Cambodia	57.079	64.234
	Nicaragua	57.159	67.127
	Morocco	58.032	32.288
	Viet Nam	59.707	79.355
	Palestine	61.234	34.904
	El Salvador	61.678	73.791
	Cabo Verde	62.188	53.507
	Republic of Moldova	62.194	108.588
	Tunisia	68.106	54.968

**Upper-middle-income**	Tuvalu	39.570	64.148
	Guyana	40.622	76.074
	Nauru	42.002	81.223
	Marshall Islands	44.004	59.089
	Turkmenistan	44.013	158.336
	Fiji	45.176	80.636
	Azerbaijan	48.181	233.396
	Saint Vincent and the Grenadines	49.494	104.678
	Samoa	49.796	36.297
	Equatorial Guinea	49.994	193.126
	Suriname	50.134	192.326
	Grenada	50.484	130.588
	Dominica	51.809	119.096
	Guatemala	52.099	71.630
	Tonga	52.417	47.056
	Dominican Republic	52.500	126.755
	Gabon	53.050	111.659
	American Samoa	53.197	114.694
	Belize	54.280	91.800
	Mauritius	55.809	122.175
	Georgia	55.953	168.837
	Jamaica	56.840	123.544
	Botswana	57.518	106.671
	Iraq	57.725	52.719
	Saint Lucia	59.140	145.745
	Kazakhstan	59.237	225.715
	South Africa	59.727	103.782
	North Macedonia	60.746	137.251
	Venezuela (Bolivarian Republic of)	60.968	89.708
	Argentina	61.158	228.643
	Mexico	61.437	191.091
	Namibia	62.169	58.533
	Armenia	62.435	204.712
	Bulgaria	62.557	291.174
	Serbia	63.349	128.223
	Paraguay	63.351	98.338
	Bosnia and Herzegovina	64.184	110.835
	Ecuador	64.454	93.193
	Brazil	64.828	153.141
	Algeria	64.855	55.946
	Sri Lanka	65.563	75.008
	Montenegro	65.959	181.352

	Libya	66.328	79.691
	Malaysia	66.574	147.259
	Maldives	66.857	161.773
	Russian Federation	68.974	296.982
	Turkey	69.210	75.532
	Iran (Islamic Republic of)	69.515	68.072
	Romania	69.585	198.340
	Albania	69.626	127.055
	China	69.712	140.232
	Jordan	69.967	90.353
	Belarus	70.462	304.461
	Thailand	71.600	144.794
	Cuba	72.592	419.935
	Colombia	74.397	133.895
	Lebanon	74.533	100.165
	Peru	75.759	121.997
	Costa Rica	79.015	130.455
**High-income**	Palau	45.067	167.348
	Saint Kitts and Nevis	52.885	237.555
	United States Virgin Islands	53.715	336.197
	Trinidad and Tobago	55.517	154.221
	Antigua and Barbuda	59.630	212.702
	Northern Mariana Islands	60.411	198.267
	Bahamas	60.565	350.806
	Barbados	61.207	233.661
	Seychelles	61.517	160.085
	United Arab Emirates	63.357	336.935
	Guam	63.773	215.371
	Saudi Arabia	64.196	172.484
	Brunei Darussalam	65.532	236.722
	Uruguay	68.530	316.401
	Greenland	68.725	303.885
	Latvia	69.788	231.087
	Lithuania	70.352	276.953
	Bahrain	70.577	204.804
	Panama	71.155	165.225
	Oman	71.221	155.776
	Hungary	72.028	210.930
	Poland	72.656	214.583
	Chile	74.350	206.290
	Puerto Rico	75.531	221.942
	Bermuda	77.549	574.848
	Slovakia	77.983	331.280
	Croatia	78.935	225.989

	Taiwan (Province of China)	79.081	161.540
	Cyprus	79.602	210.567
	Greece	80.140	168.439
	Qatar	80.403	308.650
	Israel	81.385	361.164
	Kuwait	81.833	278.052
	Czechia	81.944	317.328
	Estonia	82.039	240.494
	United States of America	82.138	440.851
	Malta	82.881	304.006
	New Zealand	82.978	471.547
	Portugal	83.533	287.959
	Denmark	84.140	671.578
	Germany	86.249	487.545
	Austria	86.370	385.455
	Belgium	87.301	373.479
	United Kingdom	87.900	467.378
	Italy	88.895	285.551
	Republic of Korea	89.162	200.518
	Australia	89.423	483.126
	Netherlands	89.588	553.078
	Slovenia	89.834	297.577
	Spain	90.006	318.583
	Canada	90.302	522.574
	Ireland	90.346	398.558
	Sweden	90.361	696.085
	France	90.766	345.967
	Finland	91.349	515.129
	Monaco	91.353	429.972
	Luxembourg	91.455	328.602
	Andorra	91.748	610.498
	Singapore	92.440	366.229
	San Marino	92.716	487.083
	Switzerland	93.498	571.659
	Norway	94.241	680.316
	Iceland	95.307	531.486
	Japan	96.341	386.805

*All health workers density (workers per 10,000 population) is identified as the indicator of health resources for health.

Table S3. The lexis diagram for the age-period-cohort model

**Period (median)**	**Age groups**														**Birth cohort (median)**
	**20-24**	**25-29**	**30-34**	**35-39**	**40-44**	**45-49**	**50-54(ref)**	**55-59**	**60-64**	**65-69**	**70-74**	**75-79**	**80-84**	**85-89**	
														X	1901-1909（1905）
													X	X	1906-1914（1910）
												X	X	X	1911-1919 (1915)
											X	X	X	X	1916-1924 (1920)
										X	X	X	X	X	1921-1929 (1925)
									X	X	X	X	X	X	1926-1934 (1930)
								X	X	X	X	X	X		1931-1939 (1935)
							X	X	X	X	X	X			1936-1944 (1940)
						X	X	X	X	X	X				1941-1949 (1945)
					X	X	X	X	X	X					**1946-1954 (1950) (ref)**
				X	X	X	X	X	X						1951-1959 (1955)
			X	X	X	X	X	X							1956-1964 (1960)
		X	X	X	X	X	X								1961-1969 (1965)
	X	X	X	X	X	X									1966-1974 (1970)
1990-1994 (1992)	X	X	X	X	X										1971-1979 (1975)
1995-1999 (1997)	X	X	X	X											1976-1984 (1980)
**2000-2004 (2002) (ref)**	X	X	X												1981-1989 (1985)
2005-2009 (2007)	X	X													1986-1994 (1990)
2010-2014 (2012)	X														1991-1999 (1995)
2015-2019 (2017)															

Note: X indicates incidence/mortality data of thyroid cancer in each age group from the corresponding period and birth cohort.

Table S4. Wald Chi-Square test for local drifts of the age-period-cohort model in four income groups

	Income Group	Gender	Wald X^2	*df*	*P*-Value
**Incidence rate**	Global	Both	195.18	14	0.00
		Male	194.39	14	0.00
		Female	117.19	14	0.00
	Low-income countries	Both	9.41	14	0.80
		Male	7.13	14	0.93
		Female	5.66	14	0.97
	Lower-middle-income countries	Both	313.61	14	0.00
		Male	95.82	14	0.00
		Female	211.22	14	0.00
	Upper-middle-income countries	Both	45.36	14	0.00
		Male	135.95	14	0.00
		Female	23.21	14	0.06
	High-income countries	Both	19.19	14	0.16
		Male	39.49	14	0.00
		Female	17.78	14	0.22
**Mortality rate**	Global	Both	51.29	14	0.00
		Male	67.96	14	0.00
		Female	35.33	14	0.00
	Low-income countries	Both	42.33	14	0.00
		Male	8.93	14	0.84
		Female	32.11	14	0.00
	Lower-middle-income countries	Both	24.33	14	0.04
		Male	3.09	14	1.00
		Female	23.42	14	0.05
	Upper-middle-income countries	Both	194.72	14	0.00
		Male	123.31	14	0.00
		Female	142.01	14	0.00
	High-income countries	Both	29.85	14	0.01
		Male	17.90	14	0.21
		Female	37.37	14	0.00

Note: The null hypothesis is all local drifts equal the net drift, which implicates temporal trends are the same in every age group.

Table S5. Wald Chi-Square test for local drifts of the age-period-cohort model in ten exemplary countries

	Income Group	Gender	Wald X^2	*df*	*P*-Value
**Incidence rate**		Both	0.96	14	1.00
	Nepal	Male	0.69	14	1.00
		Female	1.26	14	1.00
		Both	1.23	14	1.00
	Uganda	Male	1.52	14	1.00
		Female	0.97	14	1.00
		Both	66.83	14	0.00
	Viet Nam	Male	12.23	14	0.59
		Female	73.38	14	0.00
		Both	372.49	14	0.00
	India	Male	91.10	14	0.00
		Female	229.04	14	0.00
		Both	23.05	14	0.06
	China	Male	134.15	14	0.00
		Female	28.68	14	0.01
		Both	7.48	14	0.91
	Ecuador	Male	1.23	14	1.00
		Female	6.97	14	0.94
		Both	16.87	14	0.26
	Turkey	Male	2.00	14	1.00
		Female	19.99	14	0.13
		Both	44.89	14	0.00
	Korea	Male	29.68	14	0.01
		Female	47.82	14	0.00
		Both	7.66	14	0.91
	Australia	Male	5.76	14	0.97
		Female	3.63	14	1.00
		Both	36.51	14	0.00
	United States of America	Male	32.01	14	0.00
		Female	16.65	14	0.28
**Mortality rate**		Both	2.60	14	1.00
	Nepal	Male	1.64	14	1.00
		Female	1.31	14	1.00
		Both	0.78	14	1.00
	Uganda	Male	0.76	14	1.00
		Female	1.13	14	1.00
		Both	1.12	14	1.00
	Viet Nam	Male	0.43	14	1.00
		Female	0.72	14	1.00
	India	Both	37.55	14	0.00

	Male	7.80	14	0.90
	Female	24.95	14	0.04
	Both	114.17	14	0.00
China	Male	74.19	14	0.00
	Female	113.48	14	0.00
	Both	4.41	14	0.99
Ecuador	Male	2.40	14	1.00
	Female	3.34	14	1.00
	Both	12.38	14	0.58
Turkey	Male	6.75	14	0.94
	Female	10.44	14	0.73
	Both	6.65	14	0.95
Korea	Male	2.50	14	1.00
	Female	15.10	14	0.37
	Both	0.82	14	1.00
Australia	Male	1.17	14	1.00
	Female	0.72	14	1.00
	Both	8.19	14	0.88
United States of America	Male	7.03	14	0.93
	Female	3.60	14	1.00

Note: The null hypothesis is all local drifts equal the net drift, which implicates temporal trends are the same in every age group.

Table S6. Predicted number (in thousand) of thyroid cancer cases from 1990 to 2030

Year	Globe	Low-income group	Lower-middle -income group	Upper-middle -income group	High-income group
1990	88	3	17	25	42
1991	91	3	18	26	44
1992	94	3	19	27	46
1993	99	3	20	28	48
1994	103	3	21	30	49
1995	107	4	21	31	51
1996	110	4	23	32	52
1997	114	4	24	33	53
1998	119	4	25	35	55
1999	124	4	26	37	57
2000	128	4	27	38	59
2001	133	4	29	40	61
2002	138	4	30	41	63
2003	144	4	31	43	66
2004	149	4	32	45	67
2005	157	4	34	48	70
2006	162	5	36	50	72
2007	170	5	38	52	75
2008	178	5	40	56	77
2009	185	5	42	59	79
2010	191	5	44	62	80
2011	195	5	46	64	80
2012	198	6	47	65	79
2013	201	6	50	67	79
2014	205	6	50	70	78
2015	210	6	53	71	79
2016	215	7	56	73	79
2017	220	7	58	75	80
2018	227	7	61	78	81
2019	234	8	64	81	81
2020	246	8	67	83	83
2021	254	8	70	86	83
2022	262	9	73	88	84
2023	270	9	77	91	85
2024	279	10	80	94	85
2025	288	10	84	97	86
2026	297	11	88	100	87
2027	307	12	92	103	87
2028	316	12	96	106	88
2029	326	13	100	109	88
2030	337	14	105	112	89

Percentage change (%) from 2019 to 2030	44.1	76.8	64.5	38.7	9.4

Table S7. Predicted number (in thousand) of thyroid cancer deaths from 1990 to 2030

Year	Globe	Low-income group	Lower-middle-income group	Upper-middle-income group	High-income group
1990	23	1	6	7	8
1991	23	1	7	8	8
1992	24	1	7	8	8
1993	25	1	7	8	8
1994	25	1	7	8	8
1995	26	2	8	8	8
1996	26	2	8	9	8
1997	27	2	8	9	8
1998	27	2	9	9	8
1999	28	2	9	9	9
2000	29	2	9	9	9
2001	29	2	9	10	9
2002	30	2	10	10	9
2003	31	2	10	10	9
2004	31	2	10	10	9
2005	32	2	11	11	9
2006	33	2	11	11	9
2007	34	2	12	11	10
2008	35	2	12	12	10
2009	36	2	12	12	10
2010	37	2	13	13	10
2011	38	2	13	13	10
2012	39	2	13	13	10
2013	40	2	14	13	10
2014	40	2	14	14	10
2015	41	2	15	14	11
2016	42	2	15	14	11
2017	43	2	15	14	11
2018	44	2	16	15	11
2019	46	3	16	15	11
2020	47	3	17	15	11
2021	48	3	17	16	12
2022	49	3	18	16	12
2023	50	3	18	16	12
2024	52	3	19	17	12
2025	53	3	19	17	12
2026	54	3	19	17	13

2027	56	3	20	17	13
2028	57	3	20	18	13
2029	59	3	21	18	13
2030	60	4	21	18	13
Percentage change (%) from 2019 to 2030	31.7	41.2	30.4	21.7	16.6

Table S8. Age-standardized rates (per 100,000) of incidence and mortality for thyroid cancer in 2019 and 2030 and the percentage change from 2019 to 2030

	2019		2030		Percentage change (%) from 2019 to 2030	
	ASIR	ASMR	ASIR	ASMR	ASIR	ASMR
Globe	2.8	0.6	3.3	0.6	16.3	-2.7
Low-income group	1.8	0.8	2.3	0.9	30.1	9.1
Lower-middle-income group	2.3	0.7	2.9	0.7	25.1	-3.9
Upper-middle-income group	2.4	0.5	3.1	0.5	27.4	-3.1
High-income group	4.6	0.5	4.5	0.4	-2.8	-6.6

Definition of abbreviations: ASIR, age-standardized incidence rate; ASMR, age-standardized mortality rate.

Figure S1. The estimated annual percentage change of thyroid cancer age-standardized incidence rate in 31 low-income countries from 1990 to 2019

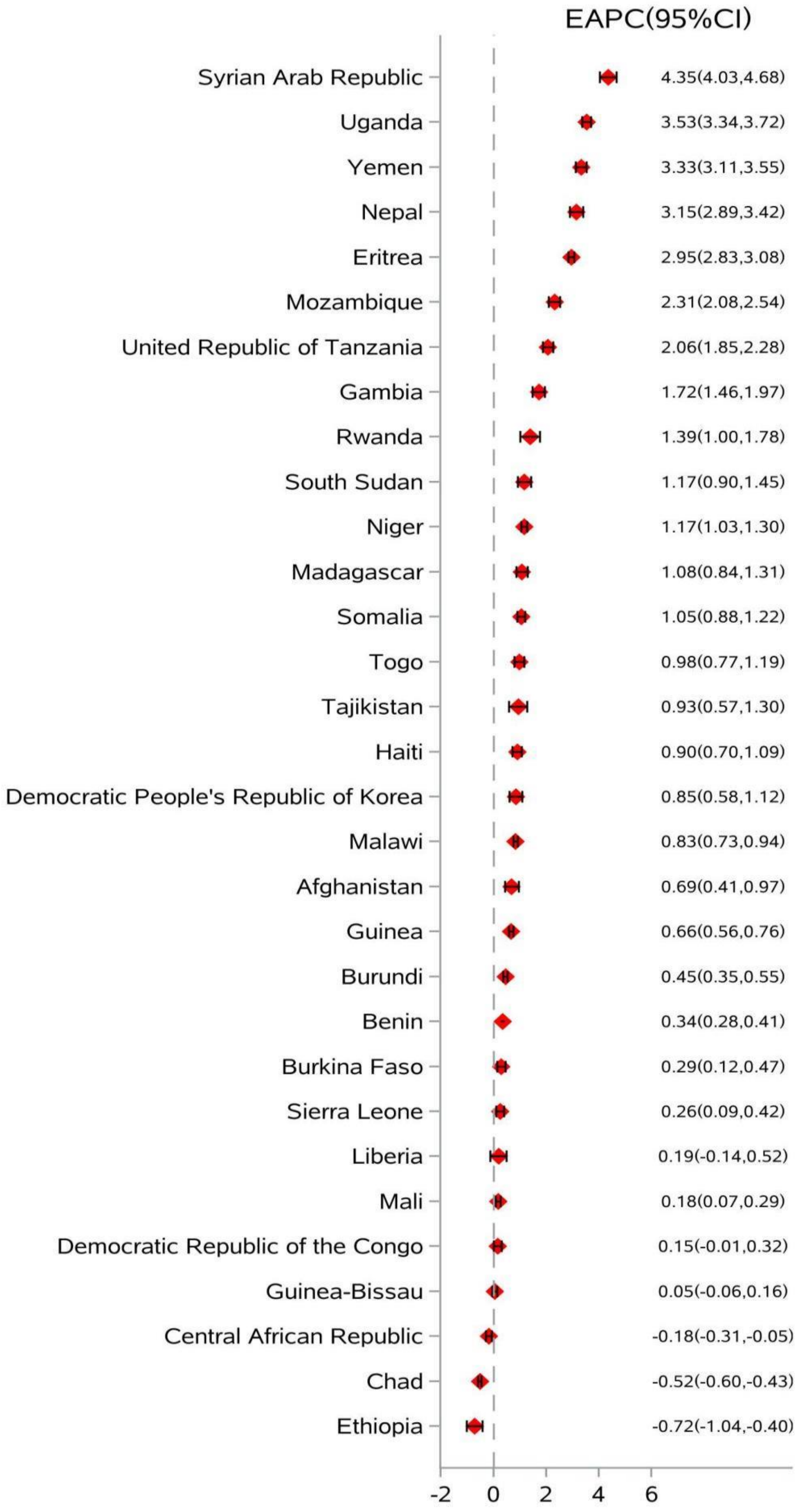

Nepal and **Uganda** were selected as exemplary countries in the low-income group.

Definition of abbreviations: EAPC, estimated annual percentage change.

Figure S2. The estimated annual percentage change of thyroid cancer age-standardized incidence rate in 47 lower-middle-income countries from 1990 to 2019

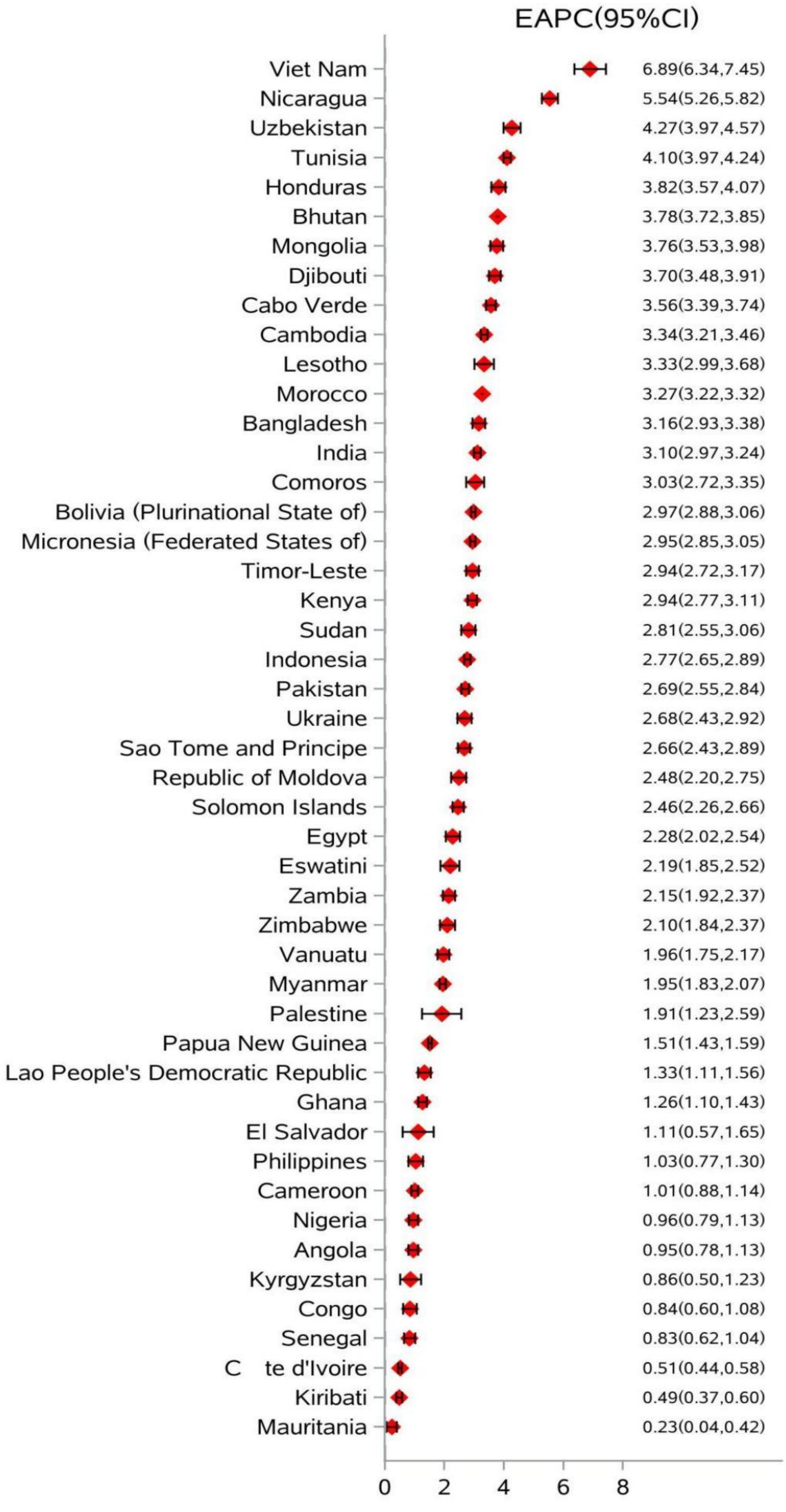

Viet Nam and **India** were selected as exemplary countries in the lower-middle-income group. Definition of abbreviations: EAPC, estimated annual percentage change.

Figure S3. The estimated annual percentage change of thyroid cancer age-standardized incidence rate in 59 upper-middle-income countries from 1990 to 2019

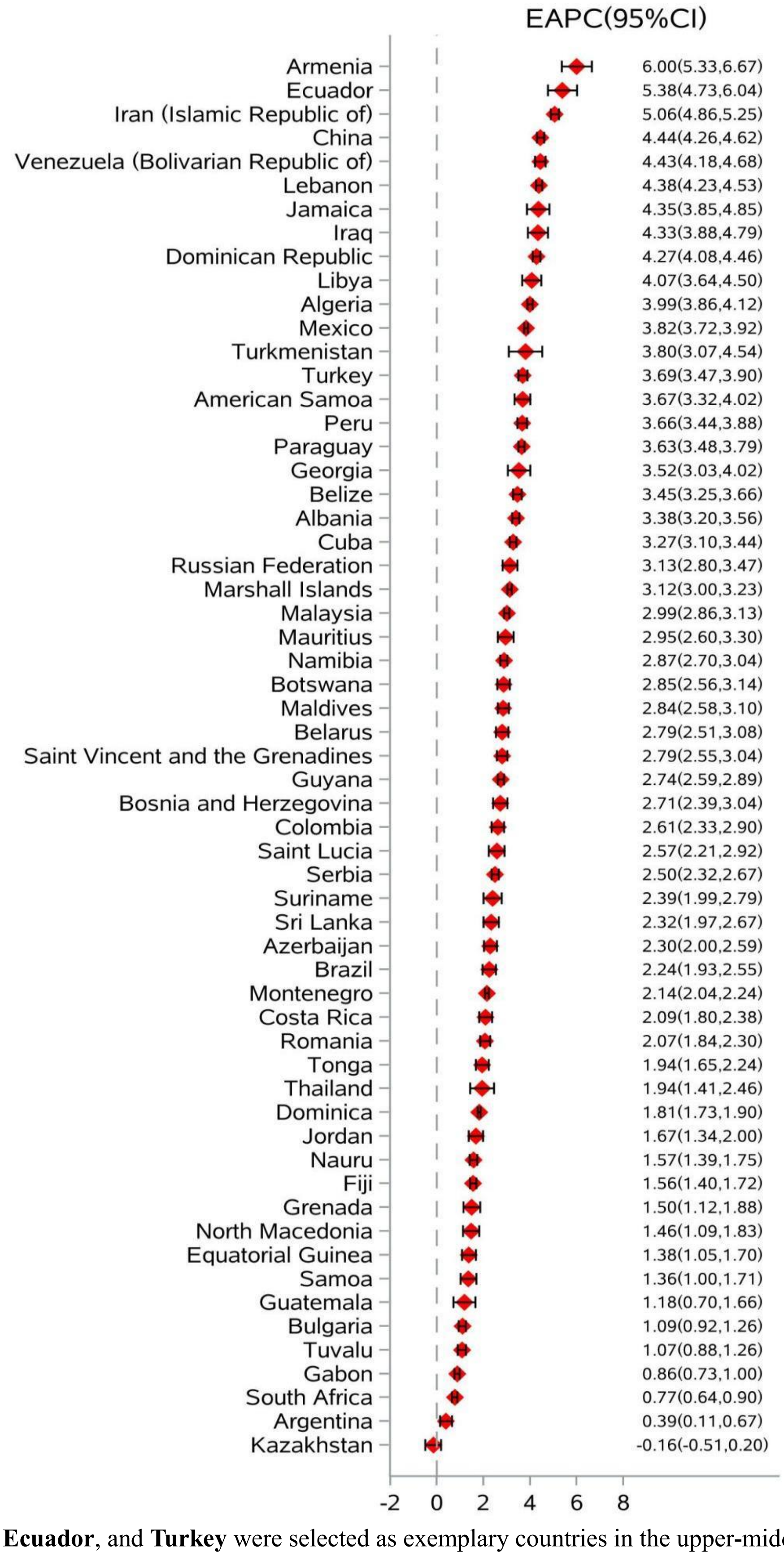

China, **Ecuador**, and **Turkey** were selected as exemplary countries in the upper-middle-income group.

Definition of abbreviations: EAPC, estimated annual percentage change.

Figure S4. The estimated annual percentage change of thyroid cancer age-standardized incidence rate in 64 high-income countries from 1990 to 2019

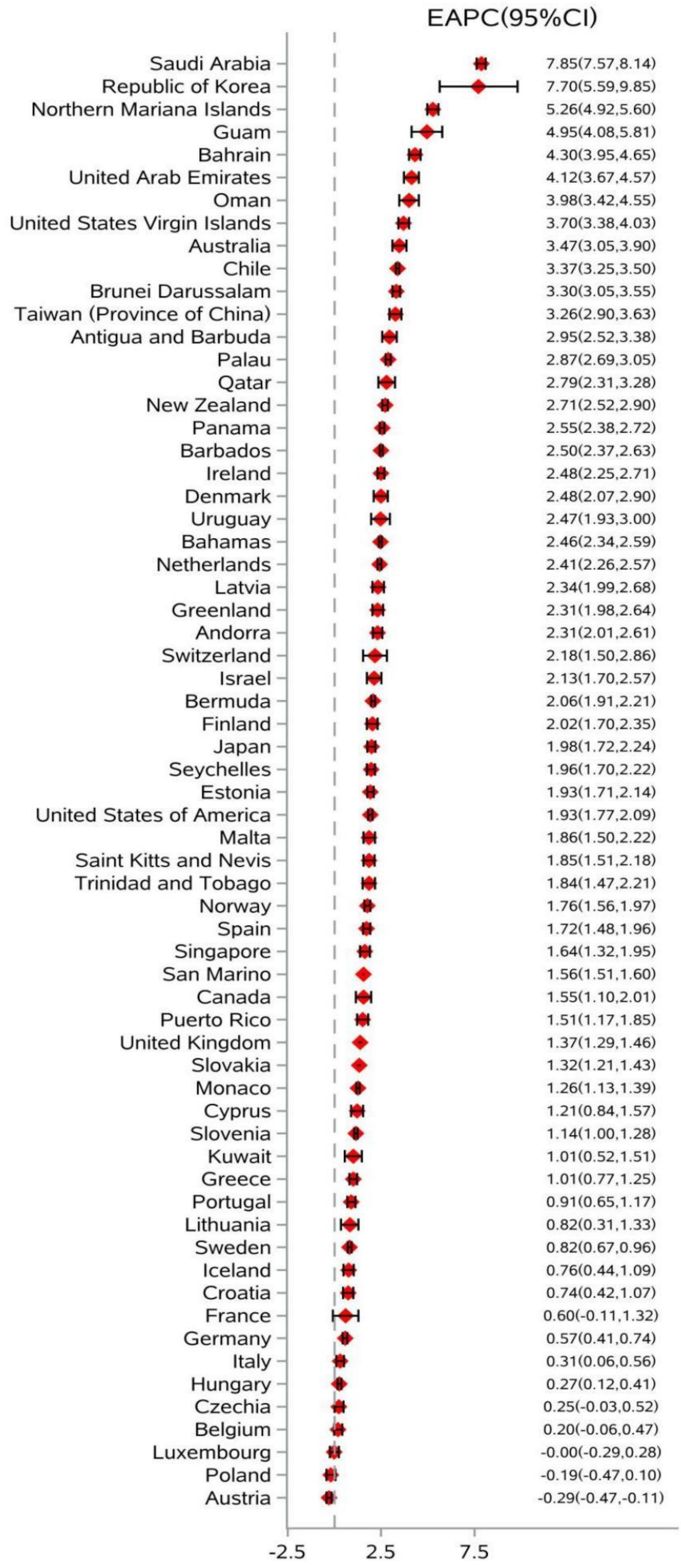

Republic of Korea, **Australia**, and **United States of American** were selected as exemplary countries in the high-income group.

Definition of abbreviations: EAPC: estimated annual percentage change.

Figure S5. Local drifts, and age, period, and cohort effects on thyroid cancer incidence and mortality in ten income-classified exemplar countries

A. Low-income countries

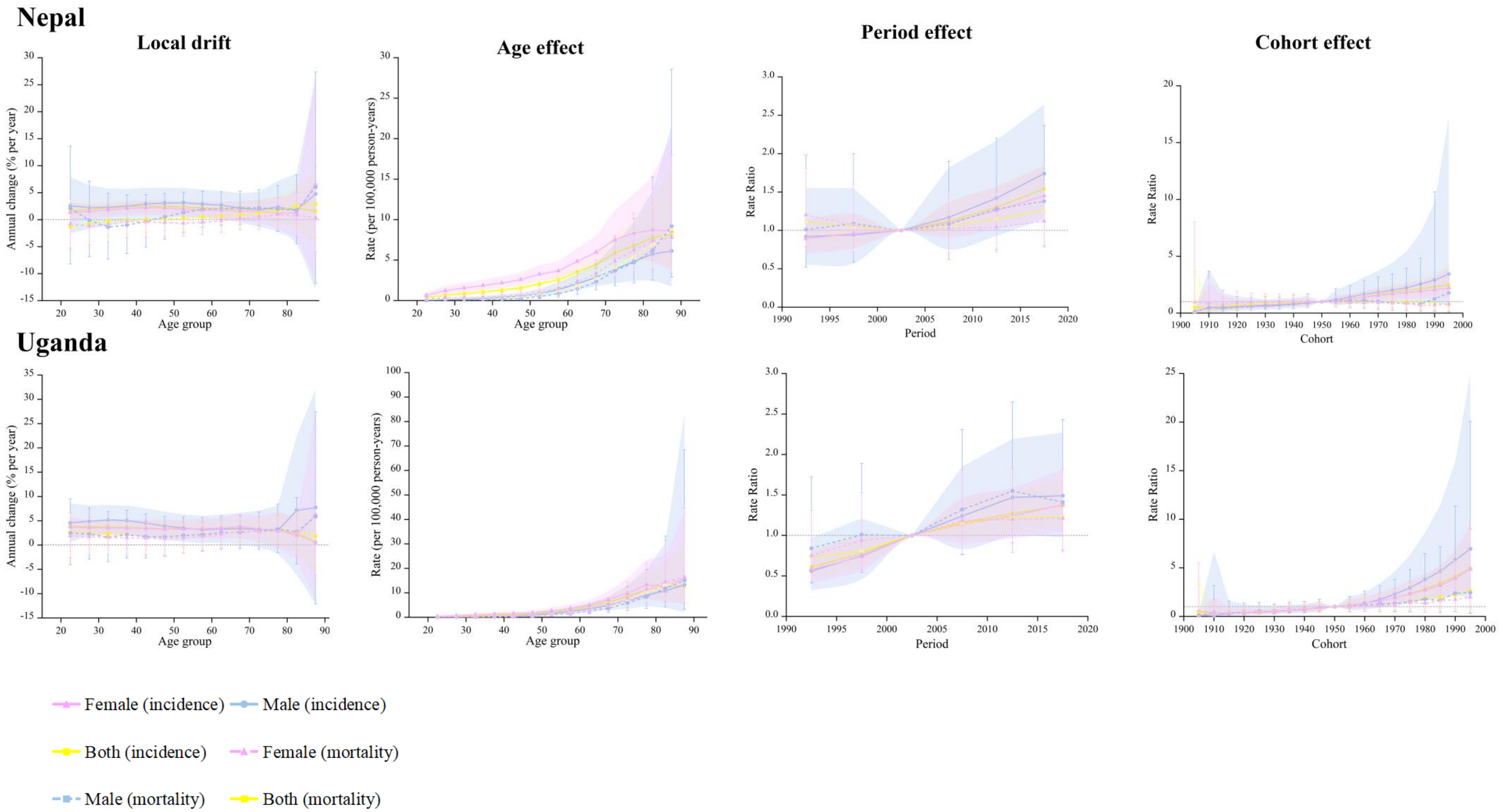

B. Lower-middle-income countries

Viet Nam

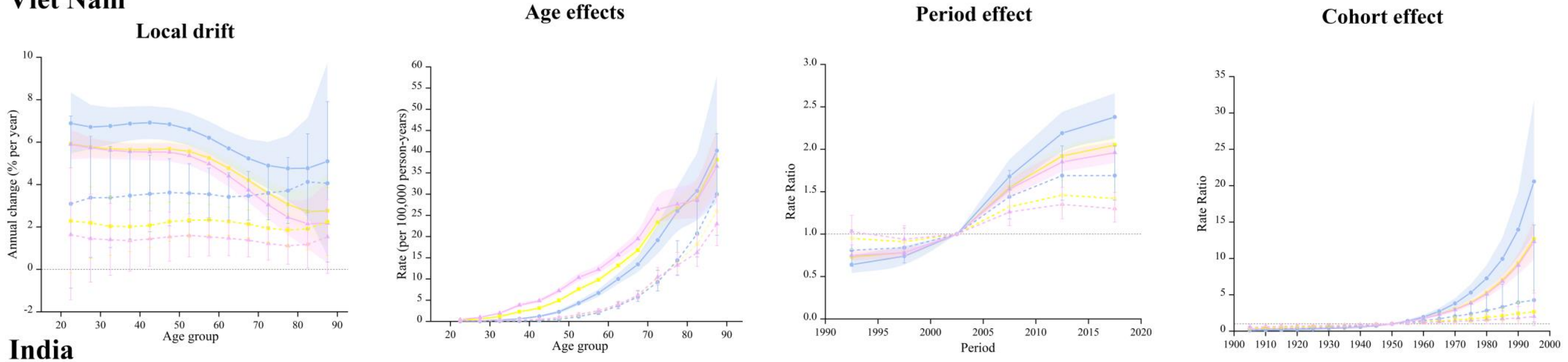

India

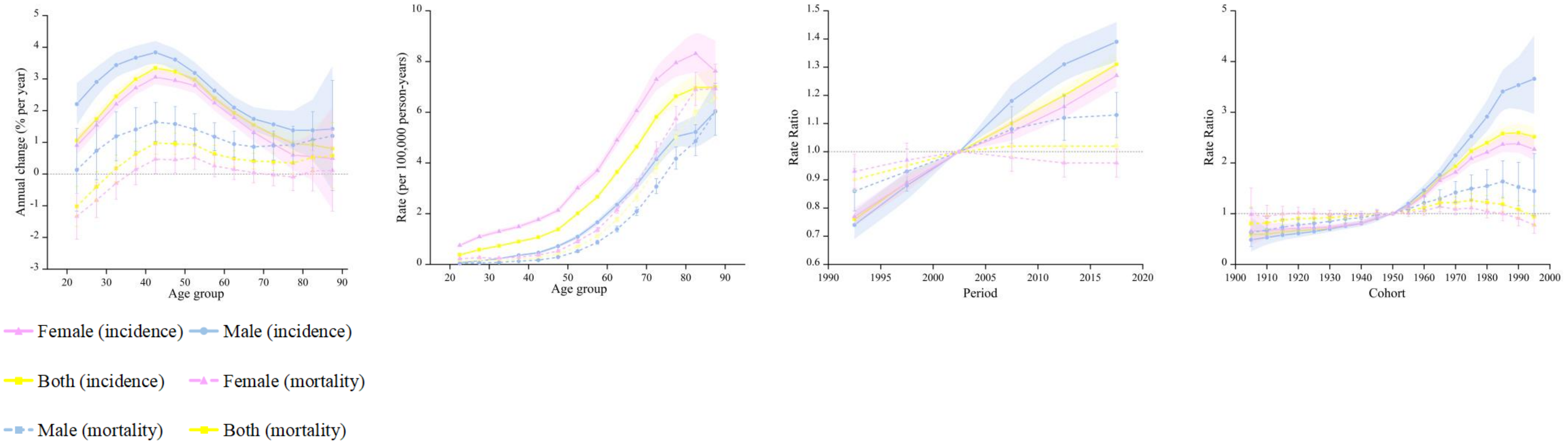

C. Upper-middle-income countries

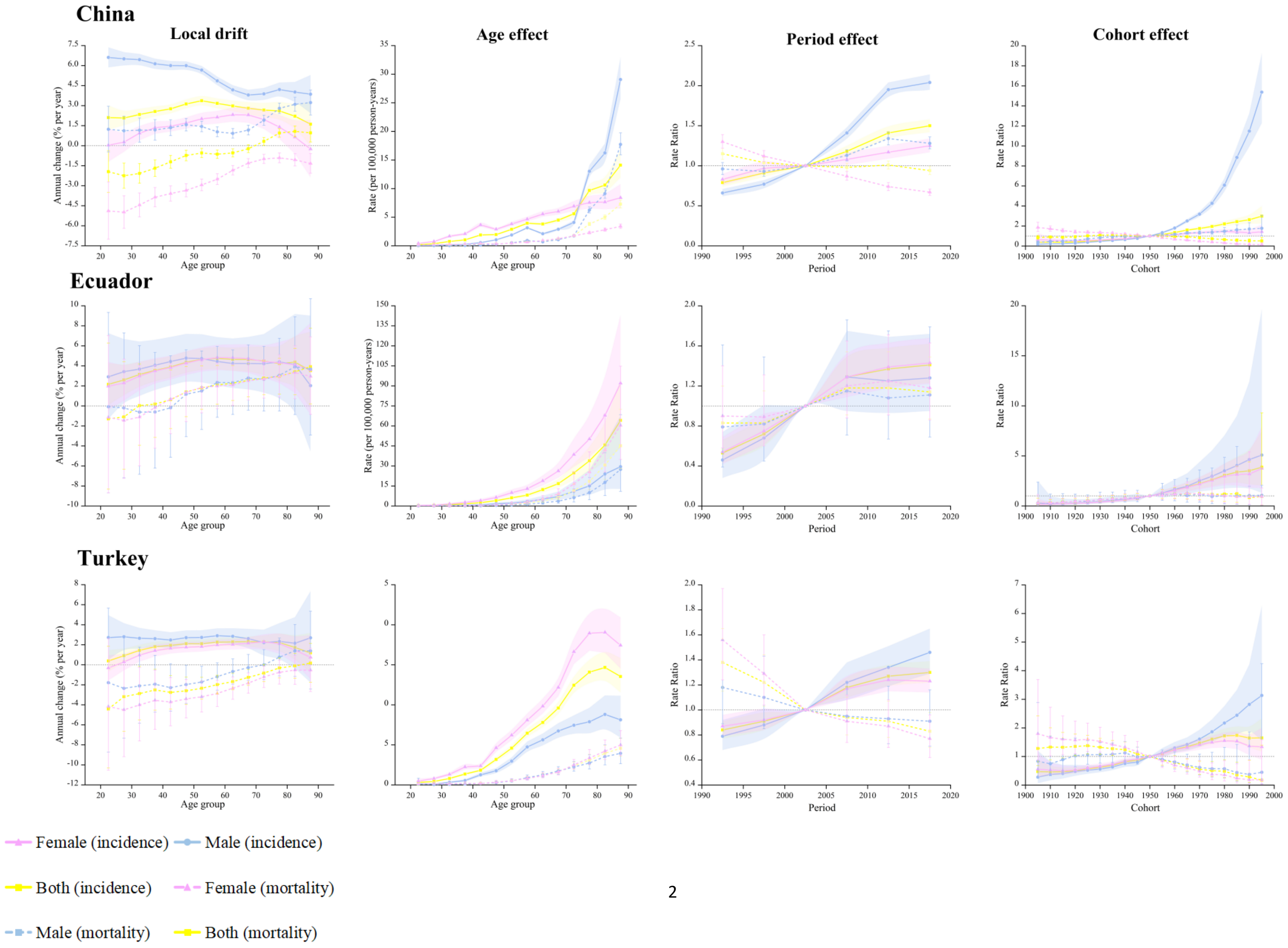

D. High-income countries

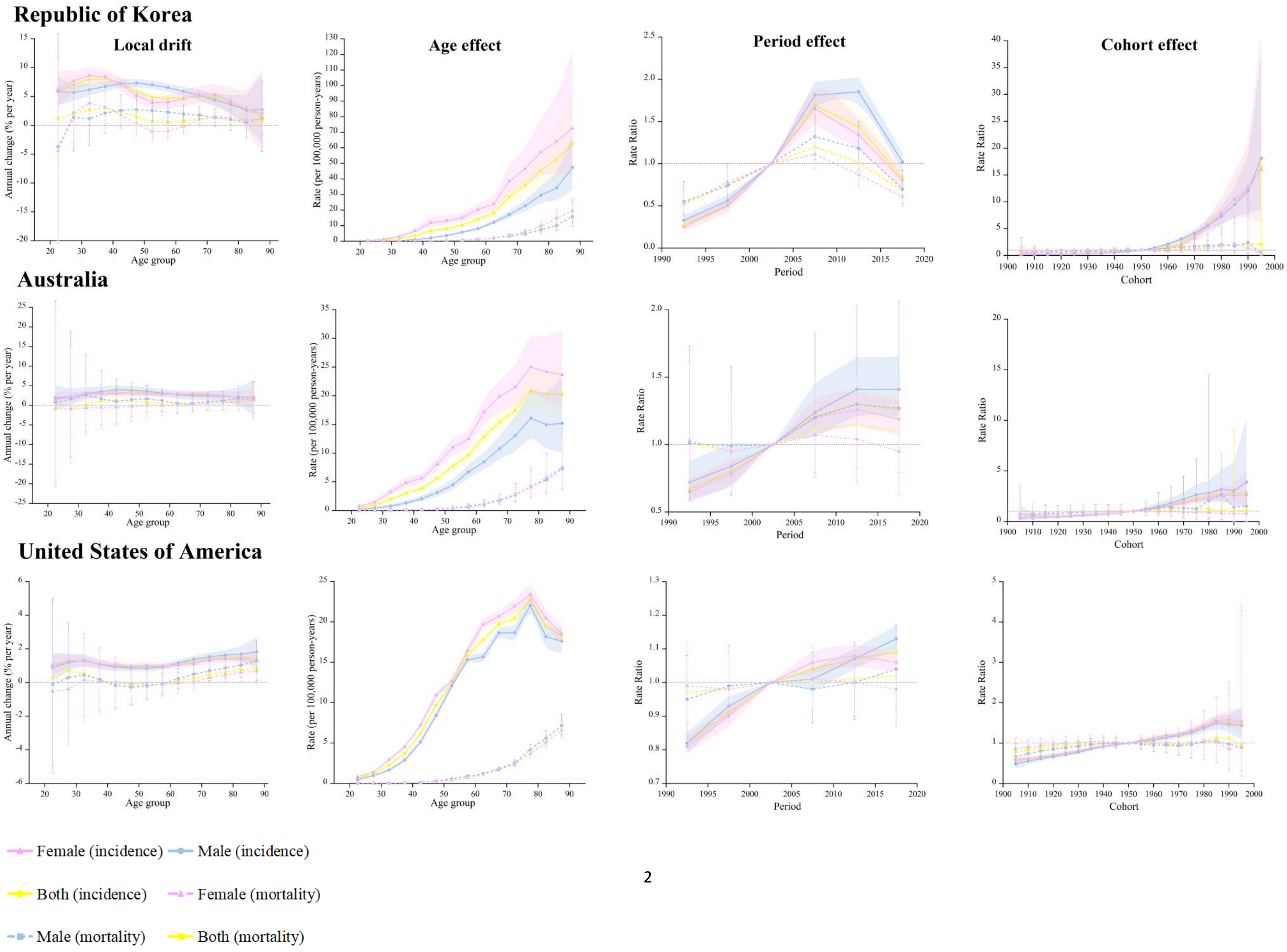

A.Local drifts of thyroid cancer incidence and mortality for 14 age groups from 1990 to 2019. B.Age effects shown by the fitted longitude age cures of thyroid cancer incidence and mortality adjusted for period deviations. C.Period effects adjusted for age and nonlinear cohort effects (calculated by the ratio of age-specific rate in each period and the reference 2000-2004 period). D.Cohort effects adjusted for age and nonlinear period effects (calculated by the ratio of age-specific rate in each cohort and the reference 1950 period)

The dots and shaded area indicate values of local drift (A), age-specific rate (B), and rate ratio (C, D), and corresponding 95% confidence intervals.

Figure S6. The temporal trends in age-standardized incidence and mortality rates of thyroid cancer from 1990 to 2019 and the prediction through 2030 in the globe and ten income-classified exemplar countries

A. Age-standardized incidence rate

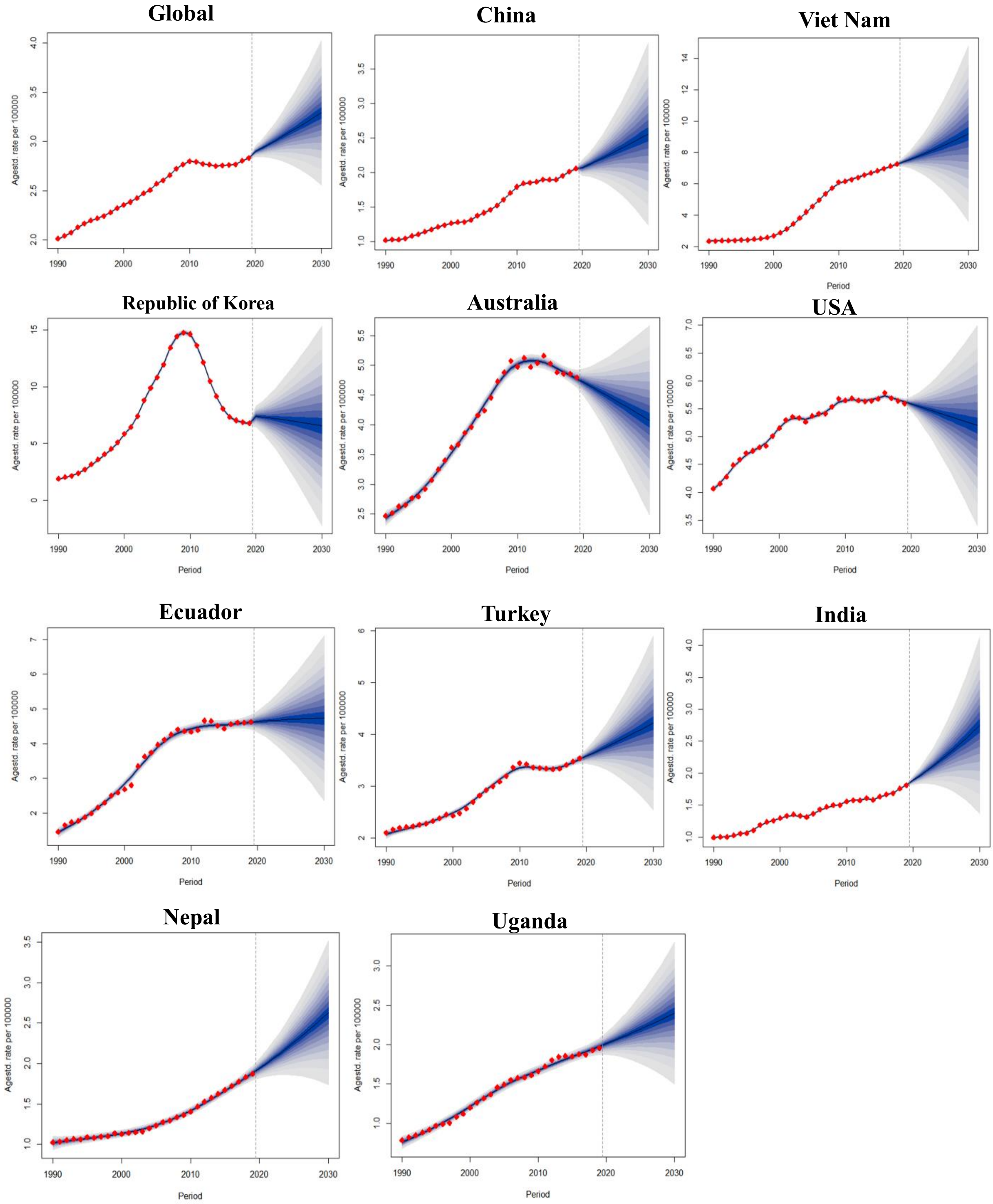

B. Age-standardized mortality rate

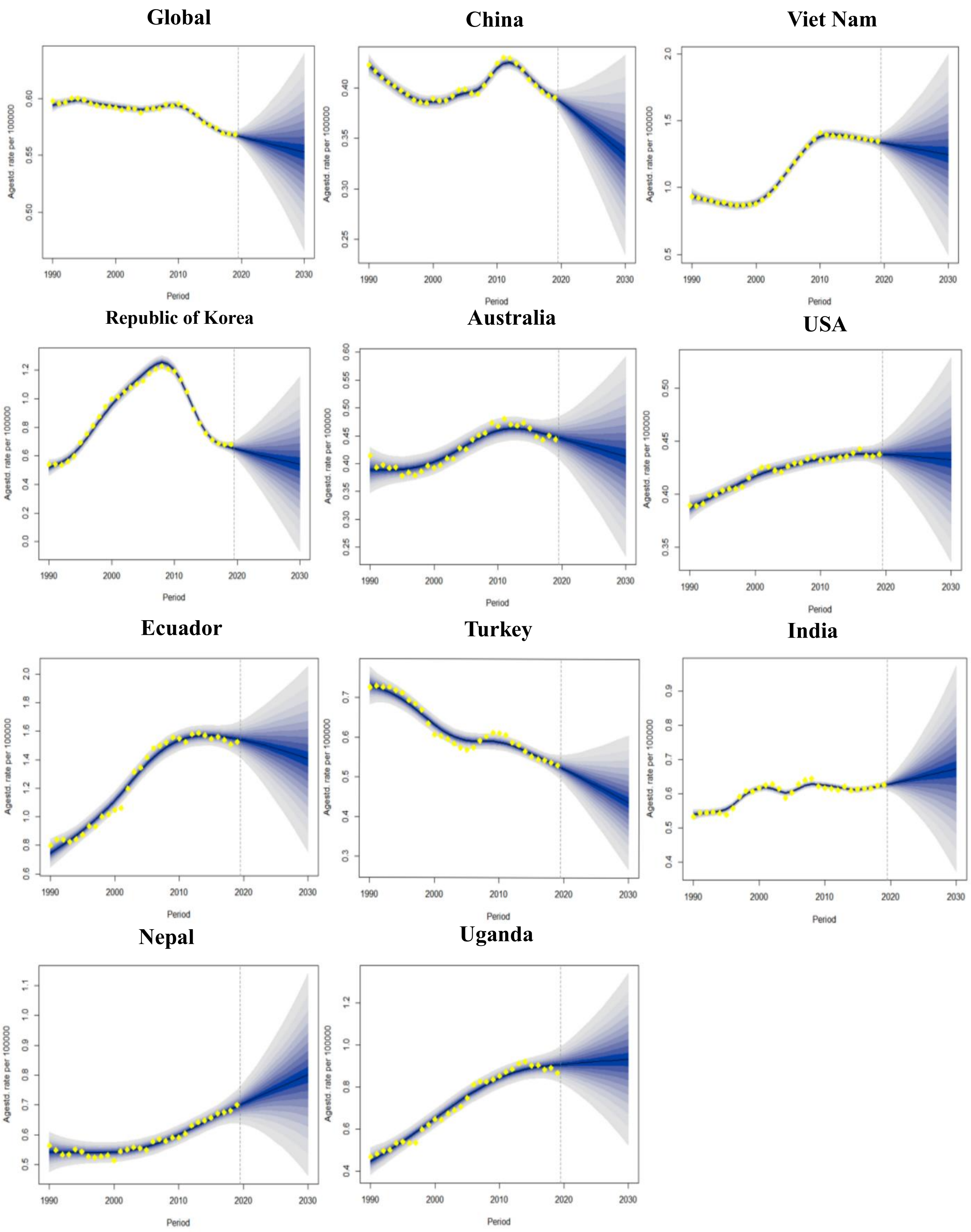

The solid dots and fan shape indicate the observed values and the predictive distribution between the 2.5 and 97.5% quantiles.

Figure S7. The temporal trends in incidence cases of thyroid cancer from 1990 to 2019 and the prediction through 2030 across age groups by income level

A. Global

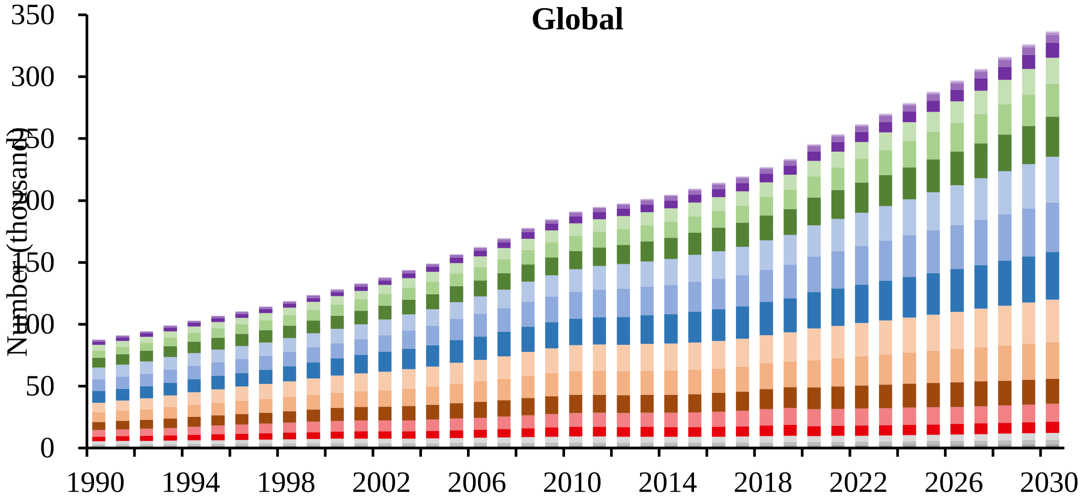

B. Low-income countries

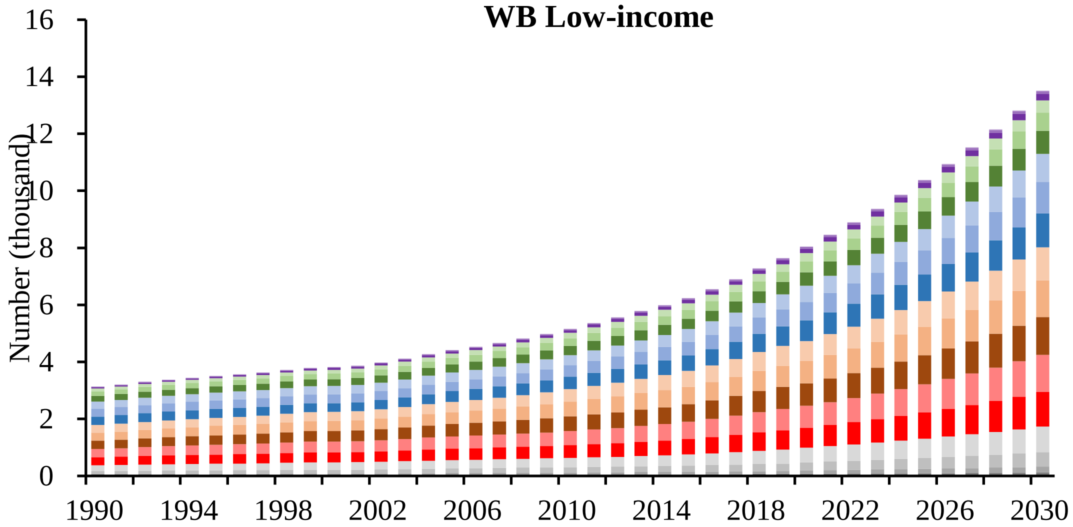

C. Lower-middle-income countries

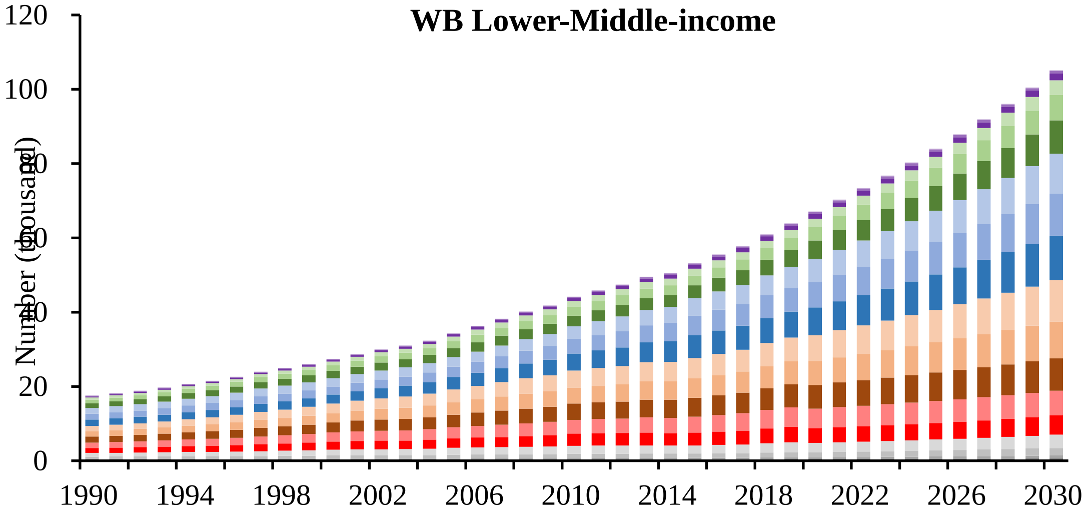

D. Upper-middle-income countries

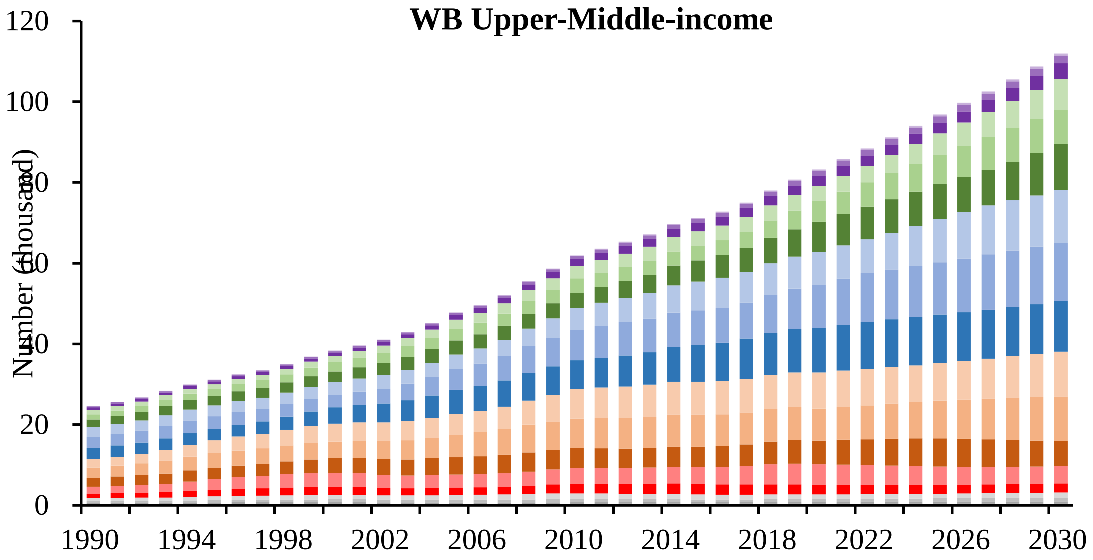

E. High-income countries

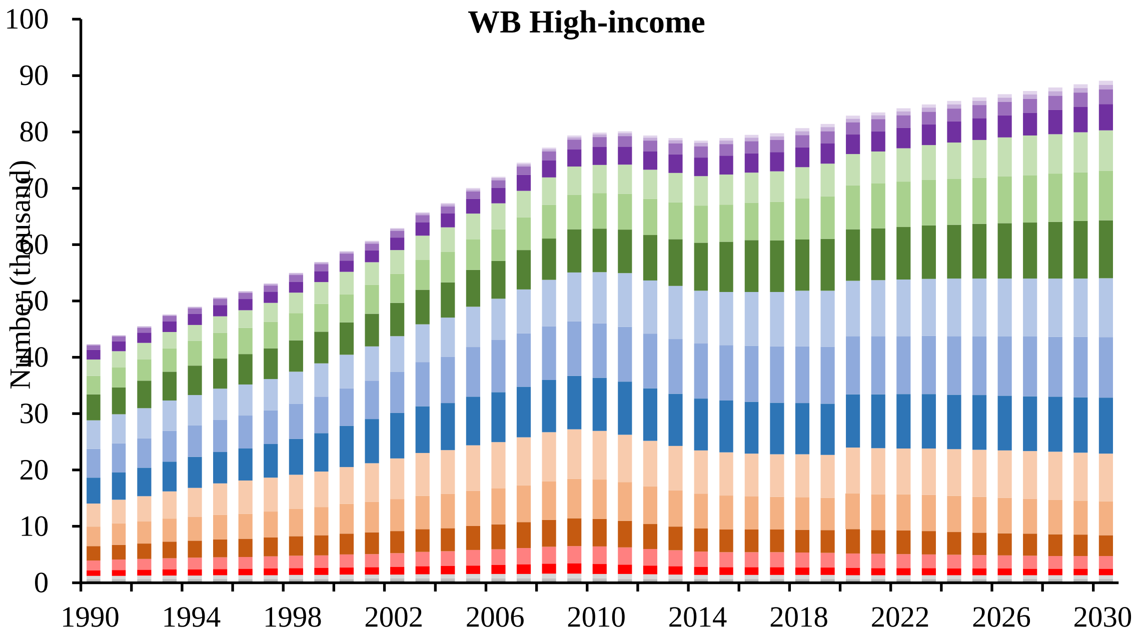

Age groups

0~4	5~9	10~14	15~19	20~24
25~29	30~34	35~39	40~44	45~49
50~54	55~59	60~64	65~69	70~74
75~79	80~84	85~89	90~94	>95

Definition of abbreviations: WB, world bank.

Figure S8. The temporal trends in death numbers of thyroid cancer from 1990 to 2019 and the prediction through 2030 across age groups by income level

A. Global

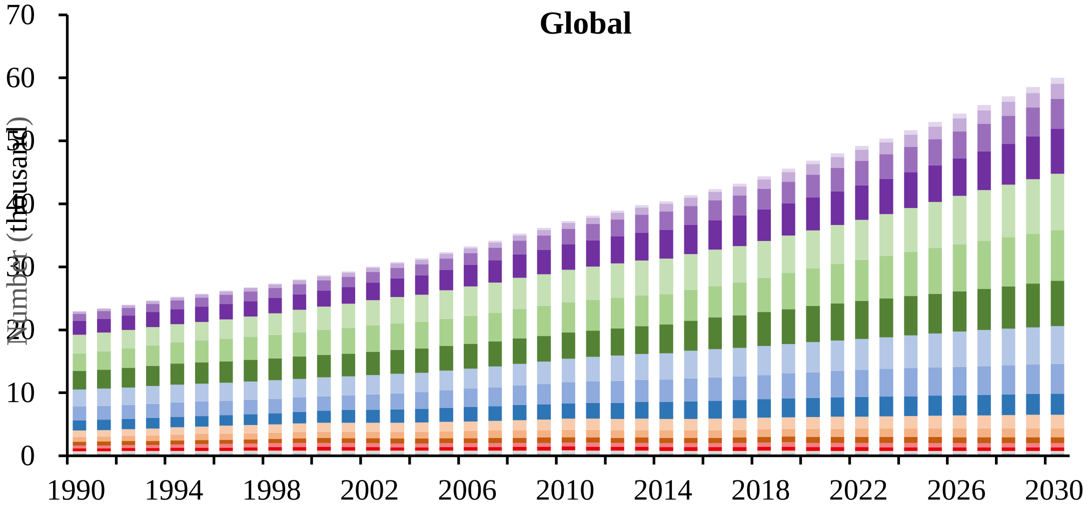

B. Low-income countries

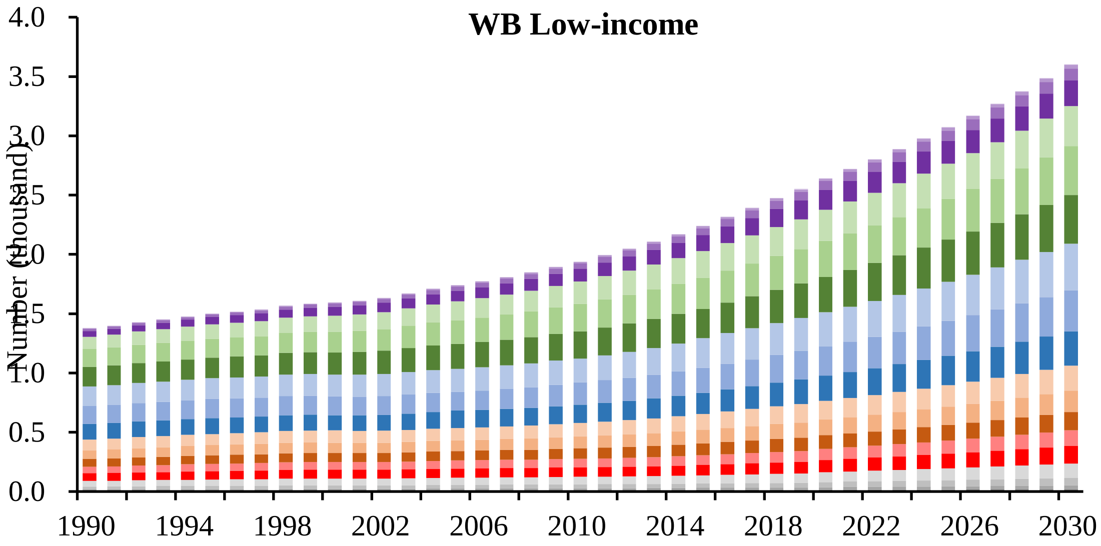

C. Lower-middle-income countries

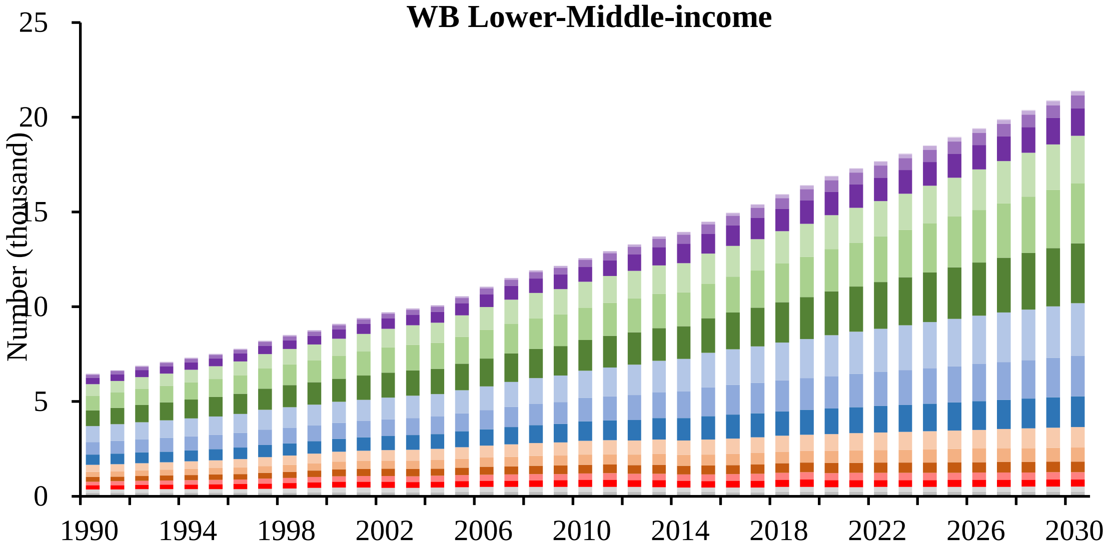
WB Lower-Middle-income
Number (thousand)
25
20
15
10
5
0
1990
1994
1998
2002
2006
2010
2014
2018
2022
2026
2030

D. Upper-middle-income countries

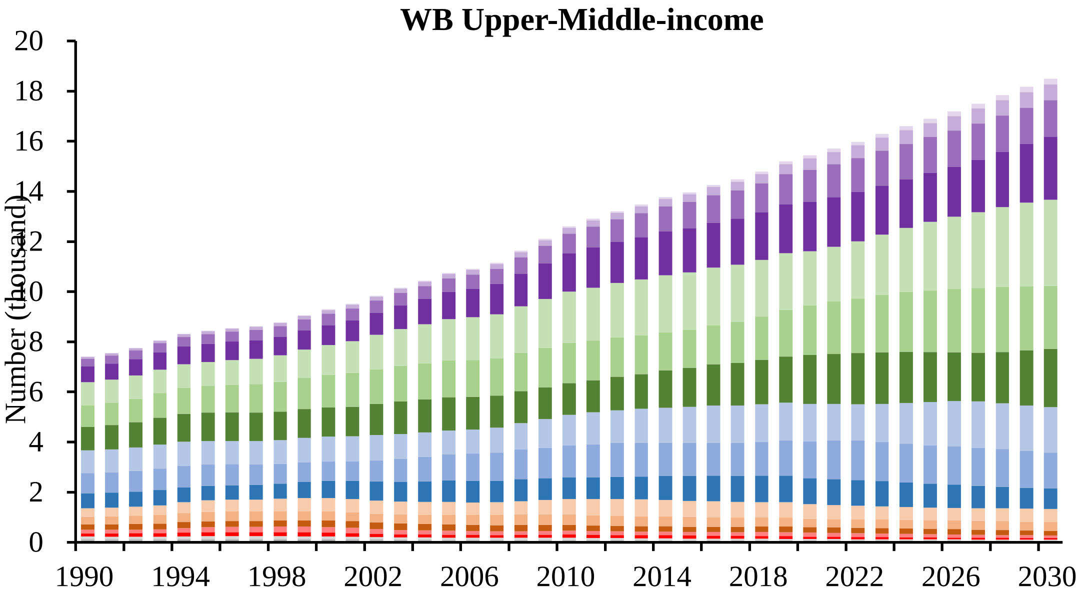

E. High-income countries

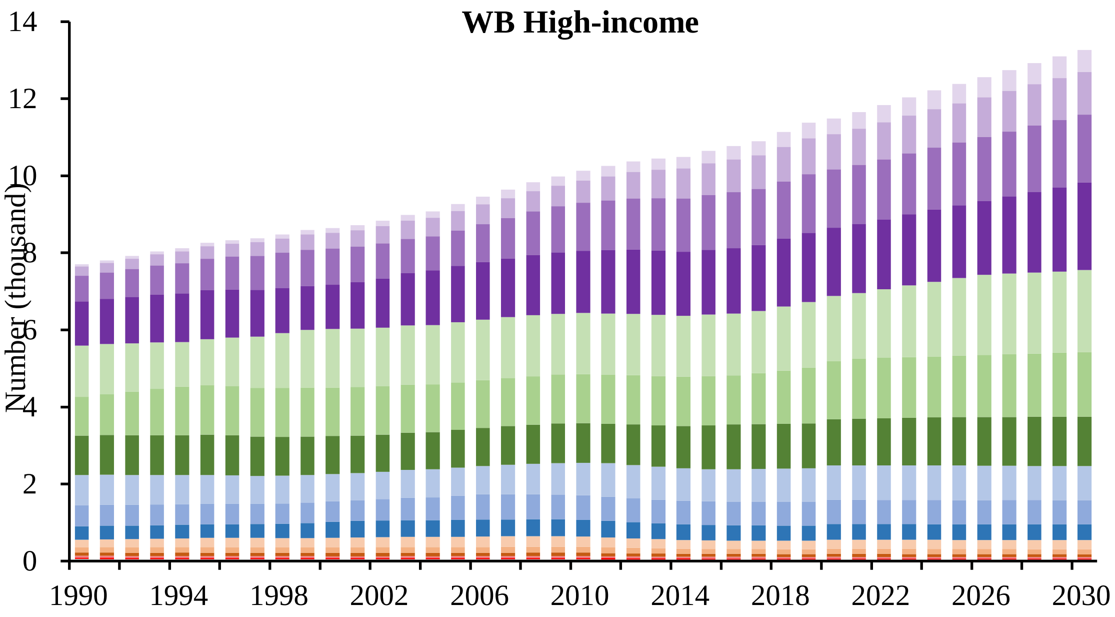

Age groups

0~4 5~9 10~14 15~19 20~24
25~29 30~34 35~39 40~44 45~49
50~54 55~59 60~64 65~69 70~74
75~79 80~84 85~89 90~94 >95

Definition of abbreviations: WB, world bank.

Figure S9. The temporal trends of age-standardized incidence rates of thyroid cancer globally by age group from 1990 to 2030

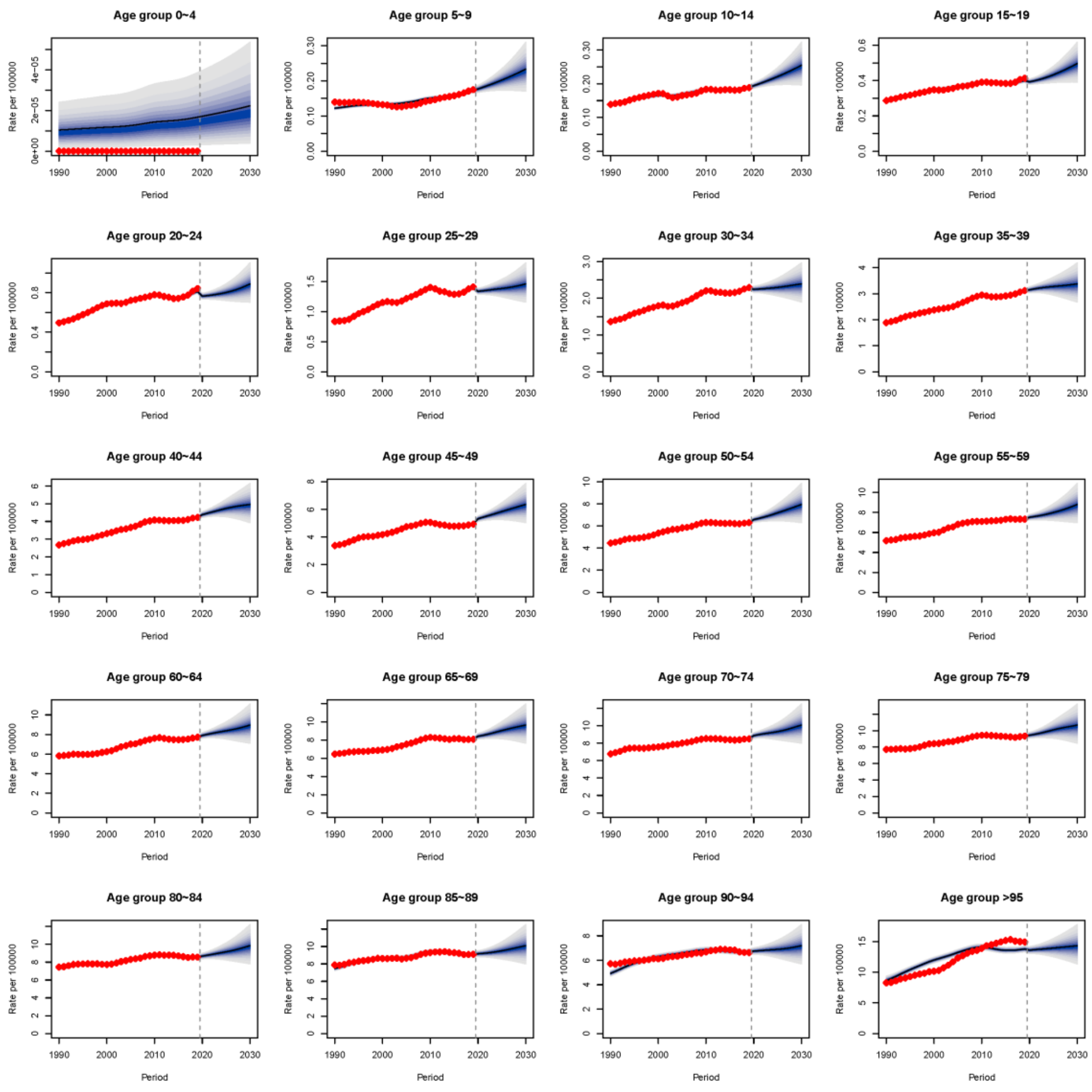

Figure S10. The temporal trends of age-standardized incidence rates of thyroid cancer in low-income countries by age group from 1990 to 2030

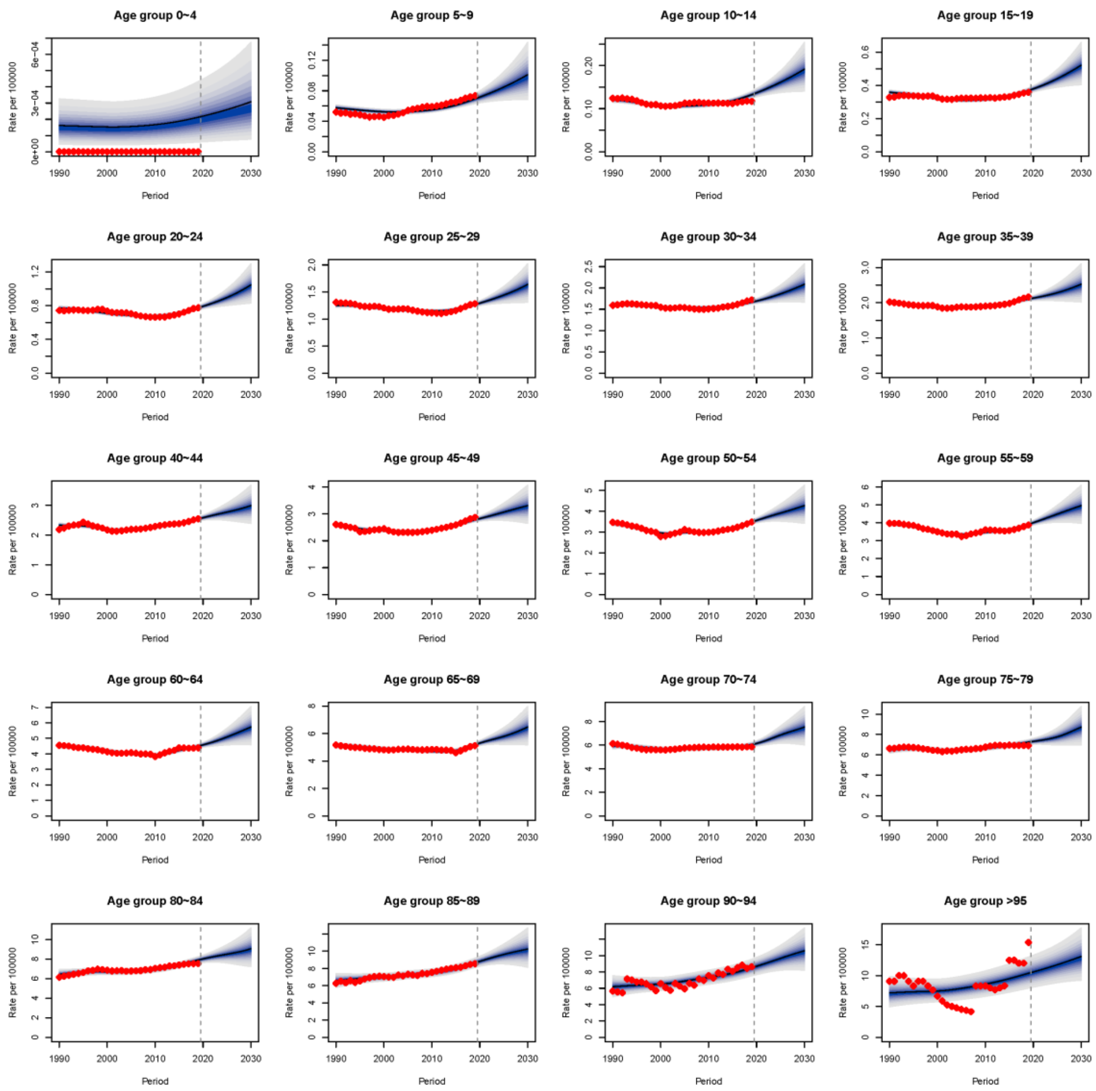

Figure S11. The temporal trends of age-standardized incidence rates of thyroid cancer in lower-middle-income countries by age group from 1990 to 2030

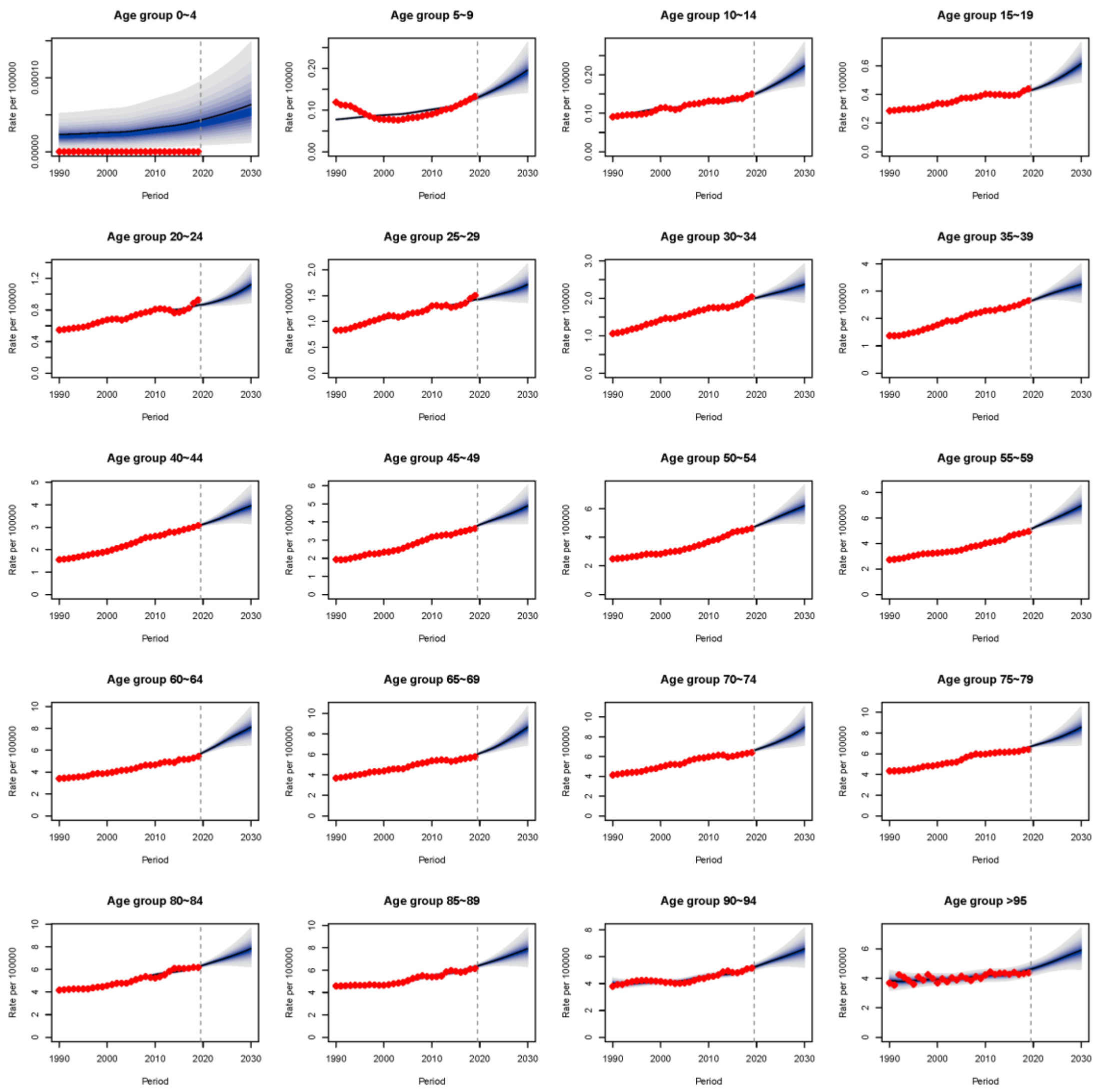

Figure S12. The temporal trends of age-standardized incidence rates of thyroid cancer in upper-middle-income countries by age group from 1990 to 2030

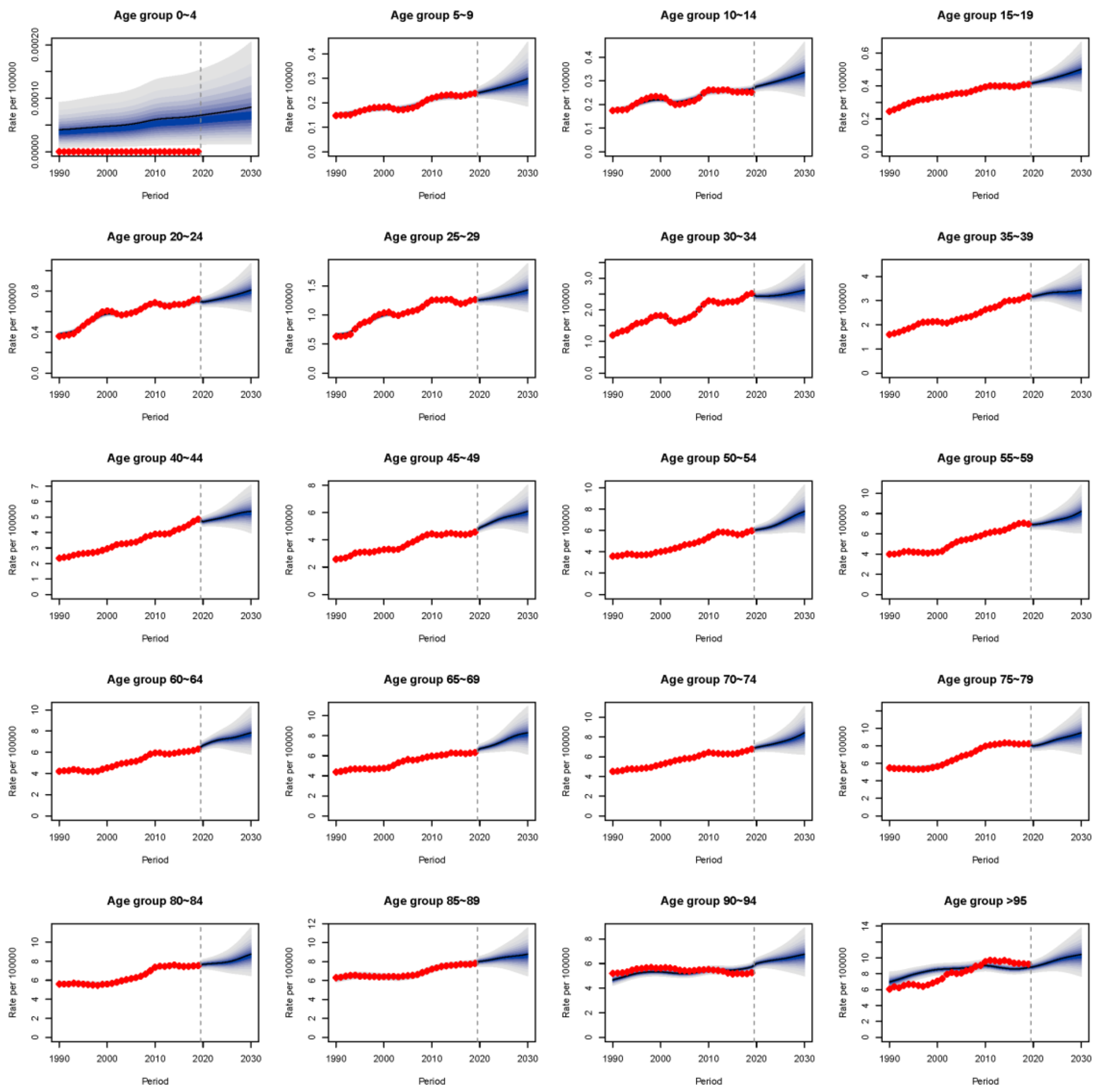

Figure S13. The temporal trends of age-standardized incidence rates of thyroid cancer in high-income countries by age group from 1990 to 2030

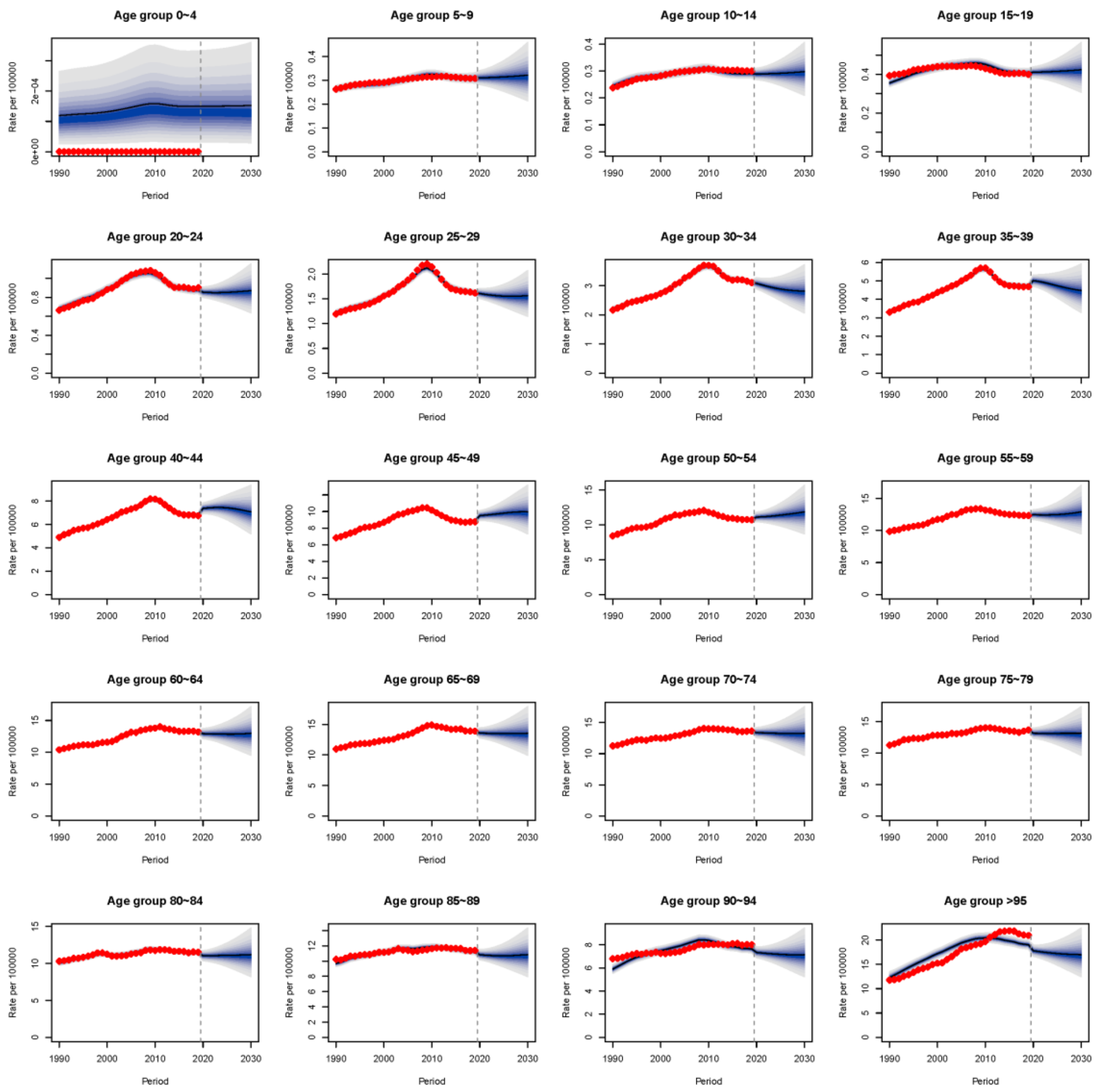

Figure S14. The temporal trends in mortality rates of thyroid cancer globally by age group from 1990 to 2030

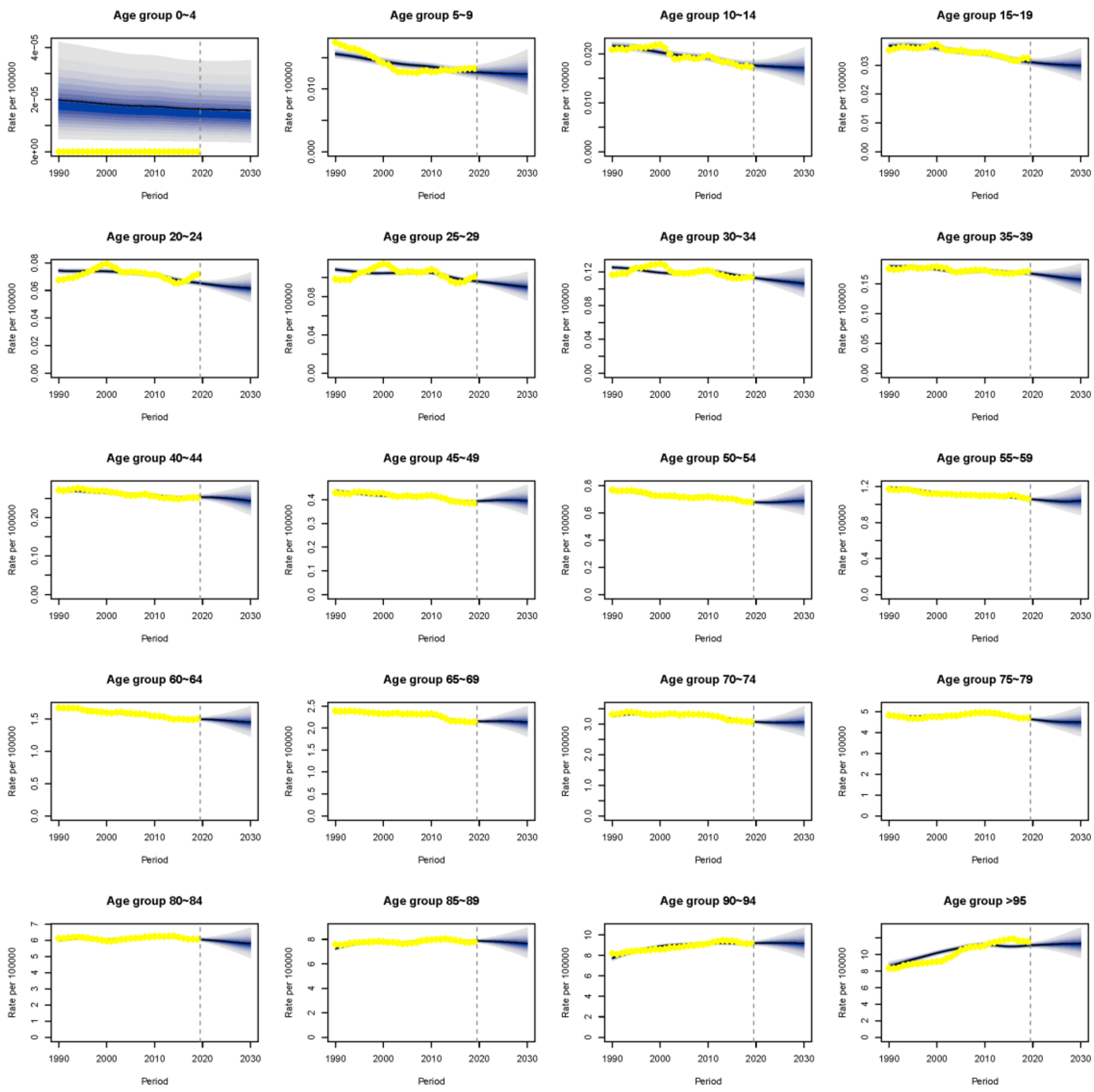

Figure S15. The temporal trends in mortality rates of thyroid cancer in low-income countries by age group from 1990 to 2030

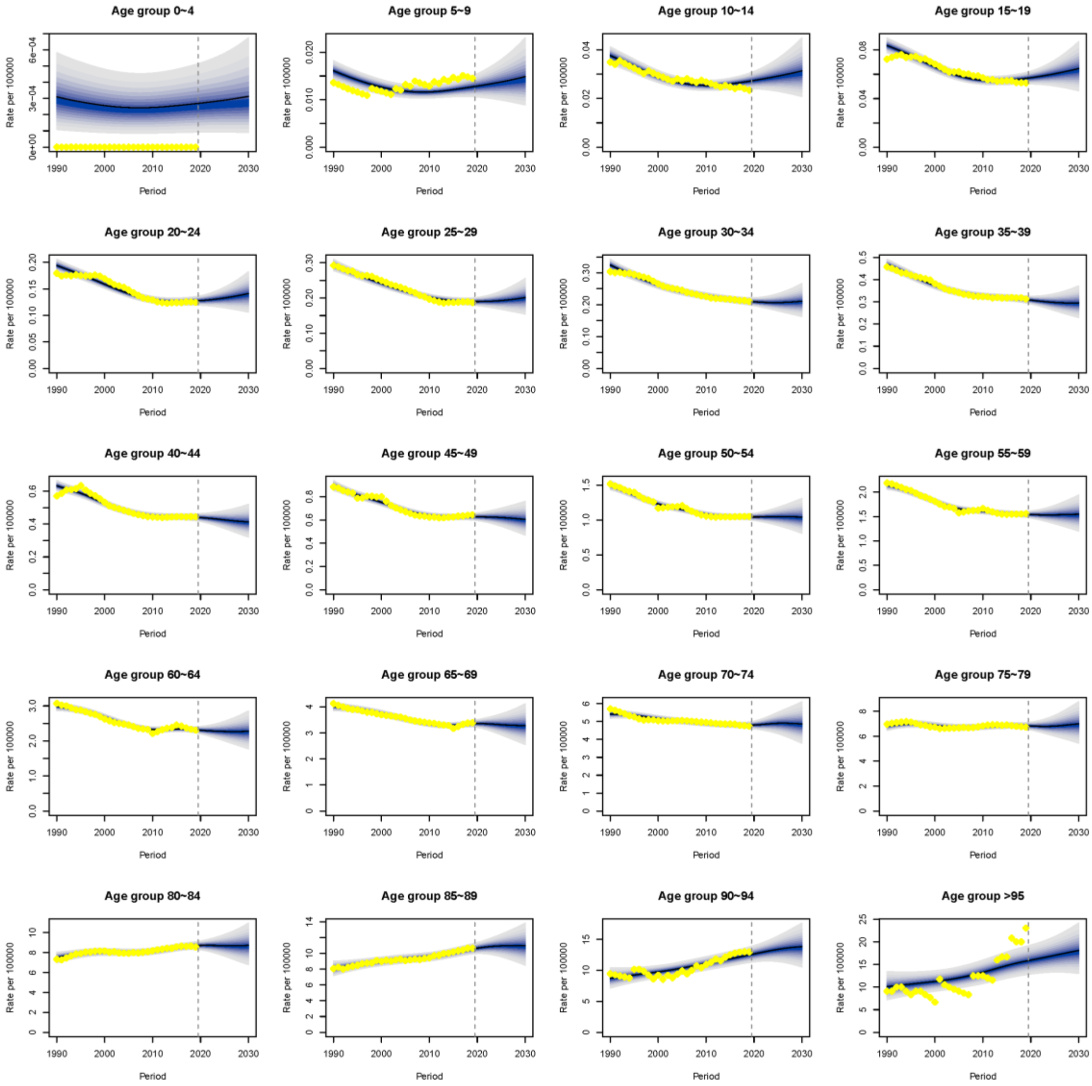

Figure S16. The temporal trends in mortality rates of thyroid cancer in lower-middle-income countries by age group from 1990 to 2030

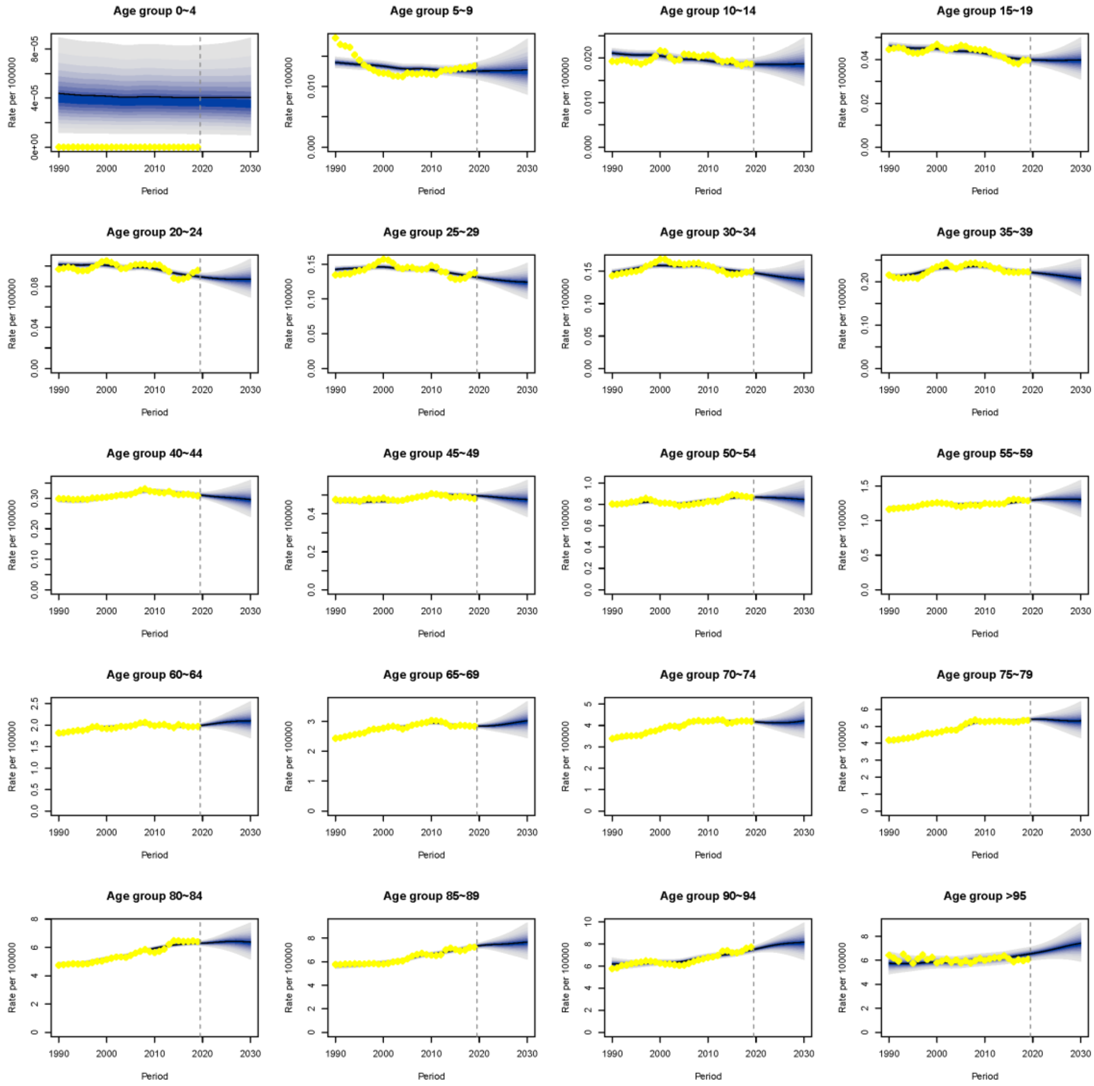

Figure S17. The temporal trends in mortality rates of thyroid cancer in upper-middle-income countries by age group from 1990 to 2030

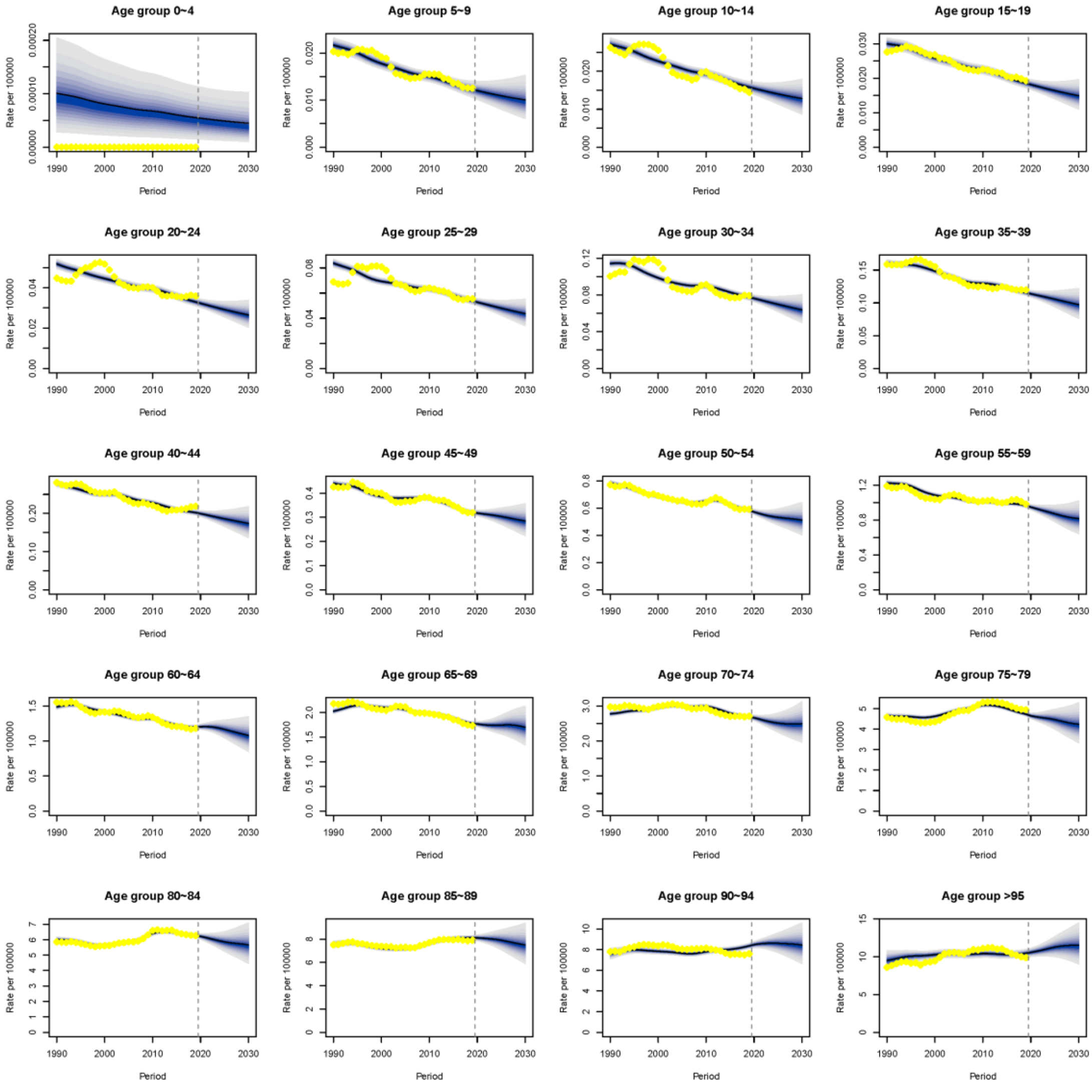

Figure S18. The temporal trends in mortality rates of thyroid cancer in high-income countries by age group from 1990 to 2030

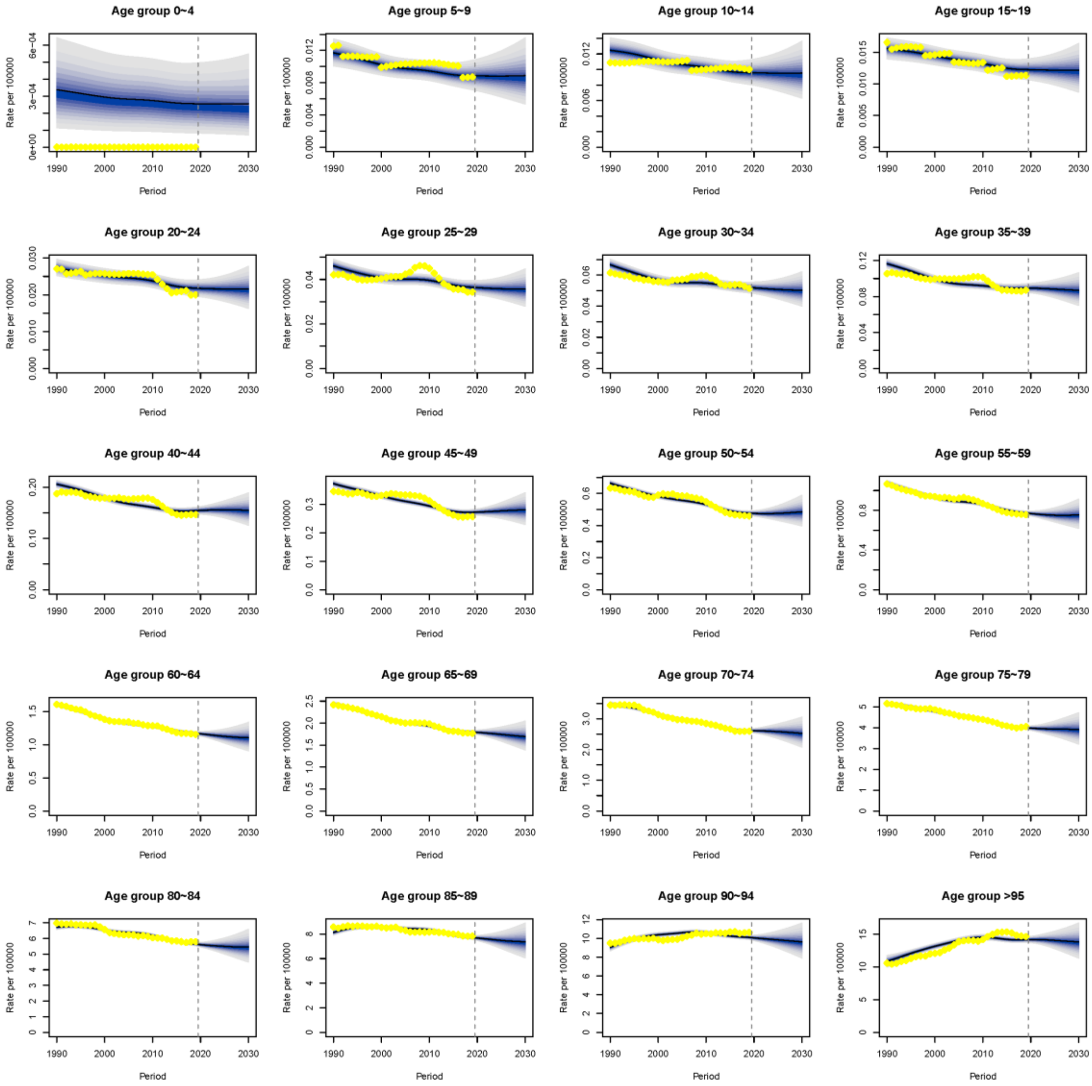

Figure S19. Association between age-standardized incidence and mortality rates of thyroid cancer and age-standardized summary exposure value of body mass index in 201 income-classified countries in 2019

A. Association between age-standardized incidence rates and BMI

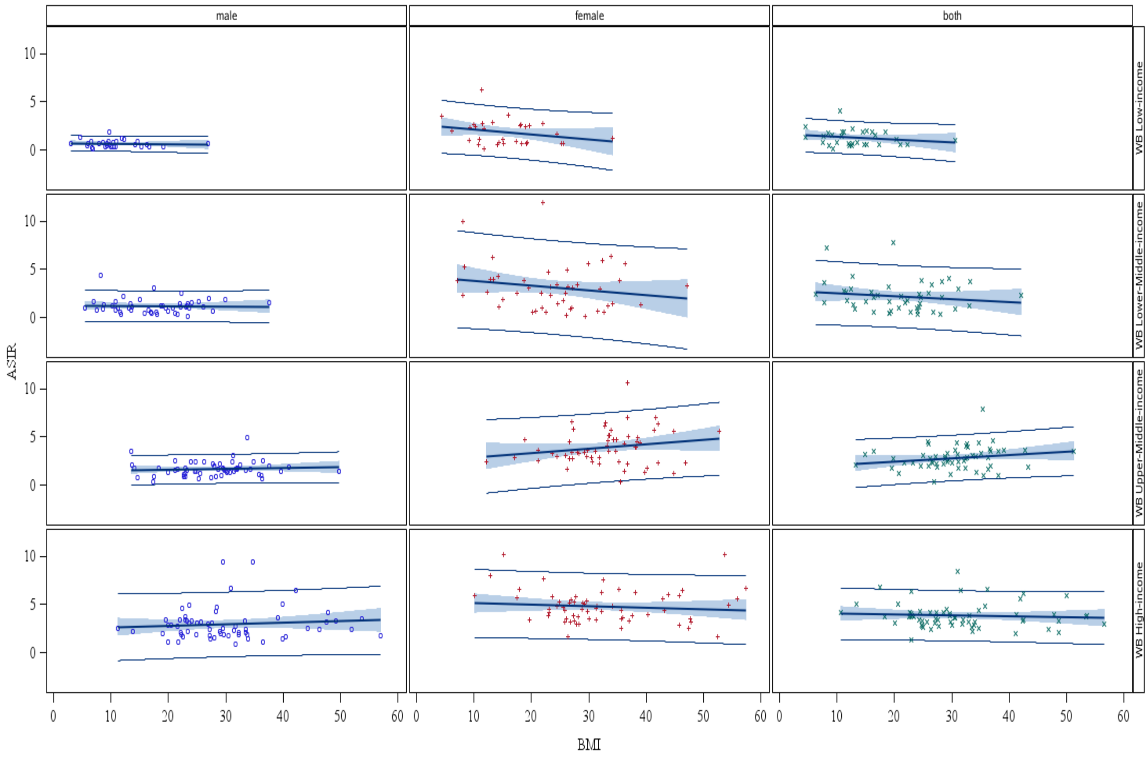

B. Association between age-standardized mortality rates and BMI

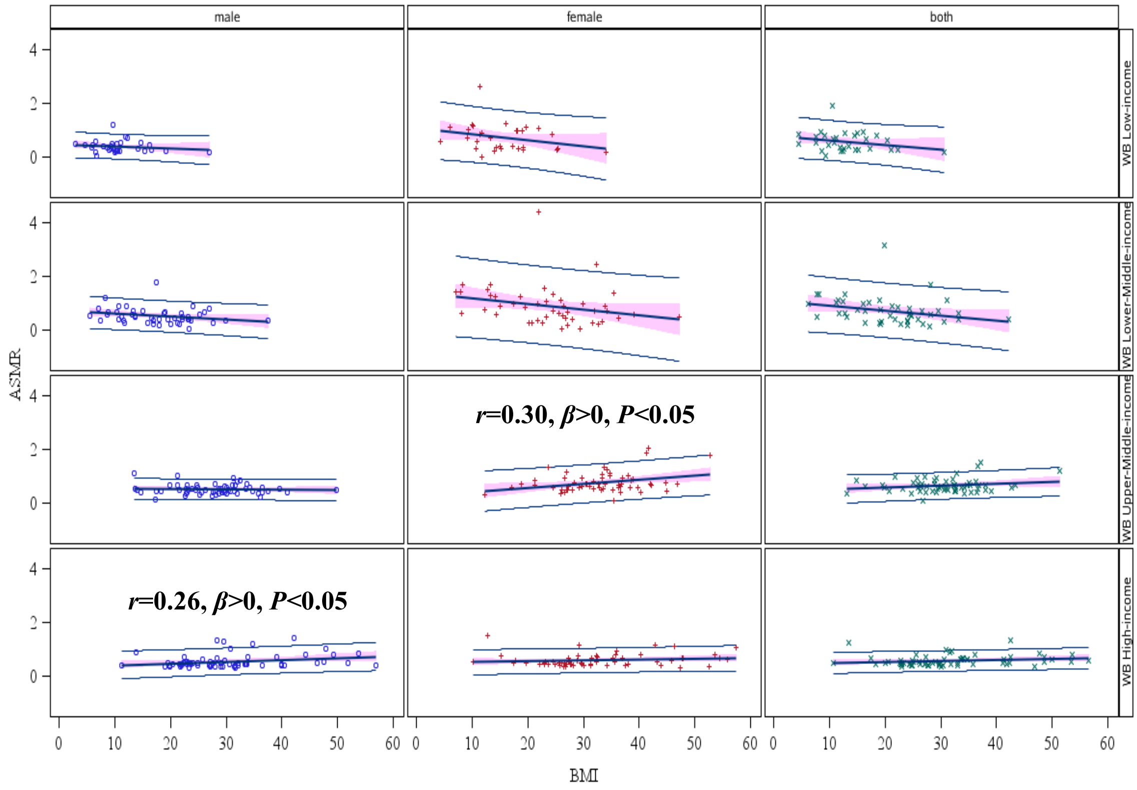

Definition of abbreviations: BMI, body mass index.

Fiure S20. Sensitivity analysis for the association between age-standardized incidence and mortality rates of thyroid cancer and universal health coverage in 201 SDI-classified countries in 2019

A. Association between age-standardized incidence rates and universal health coverage

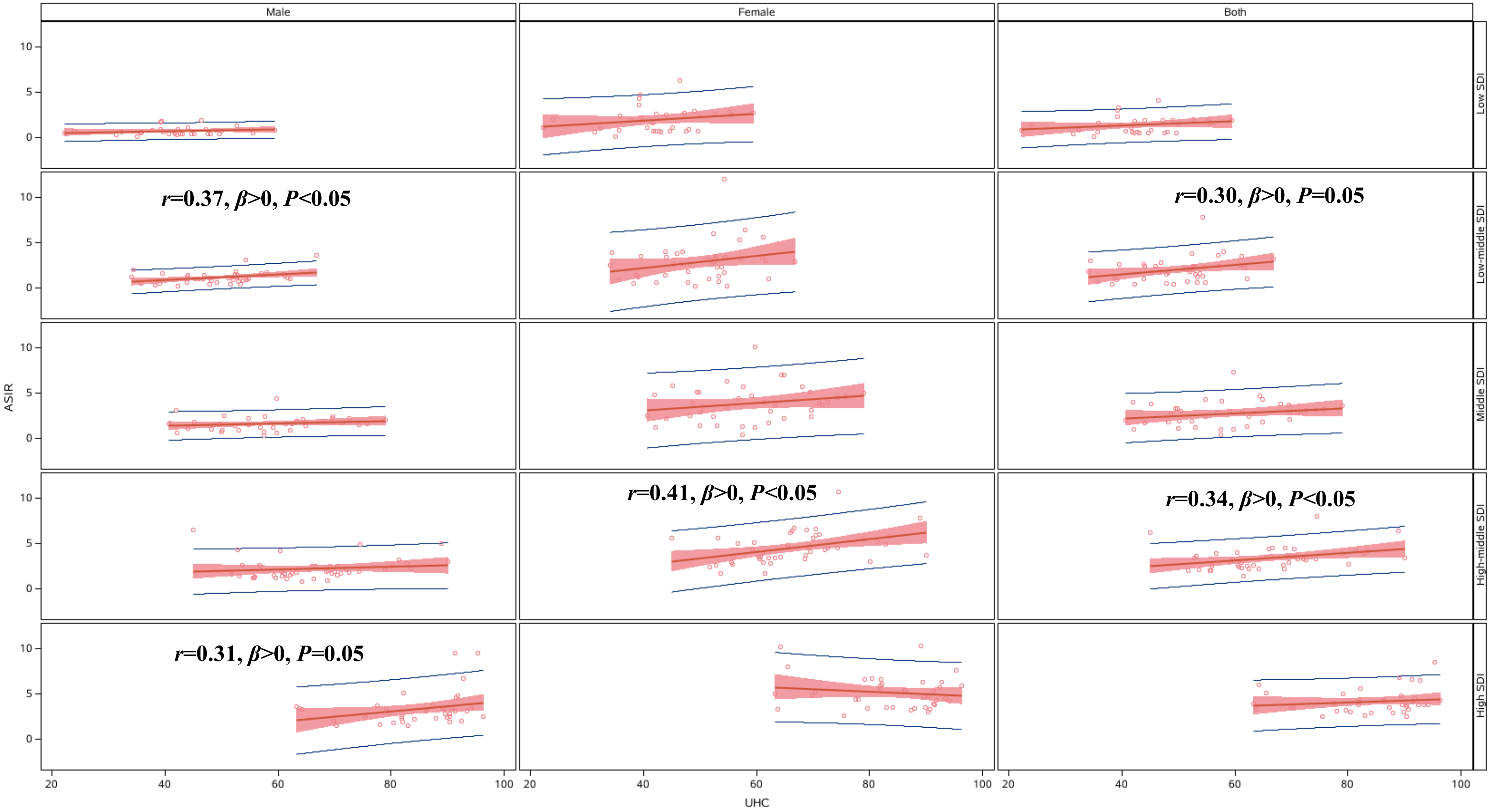

B. Association between age-standardized mortality rates and universal health coverage

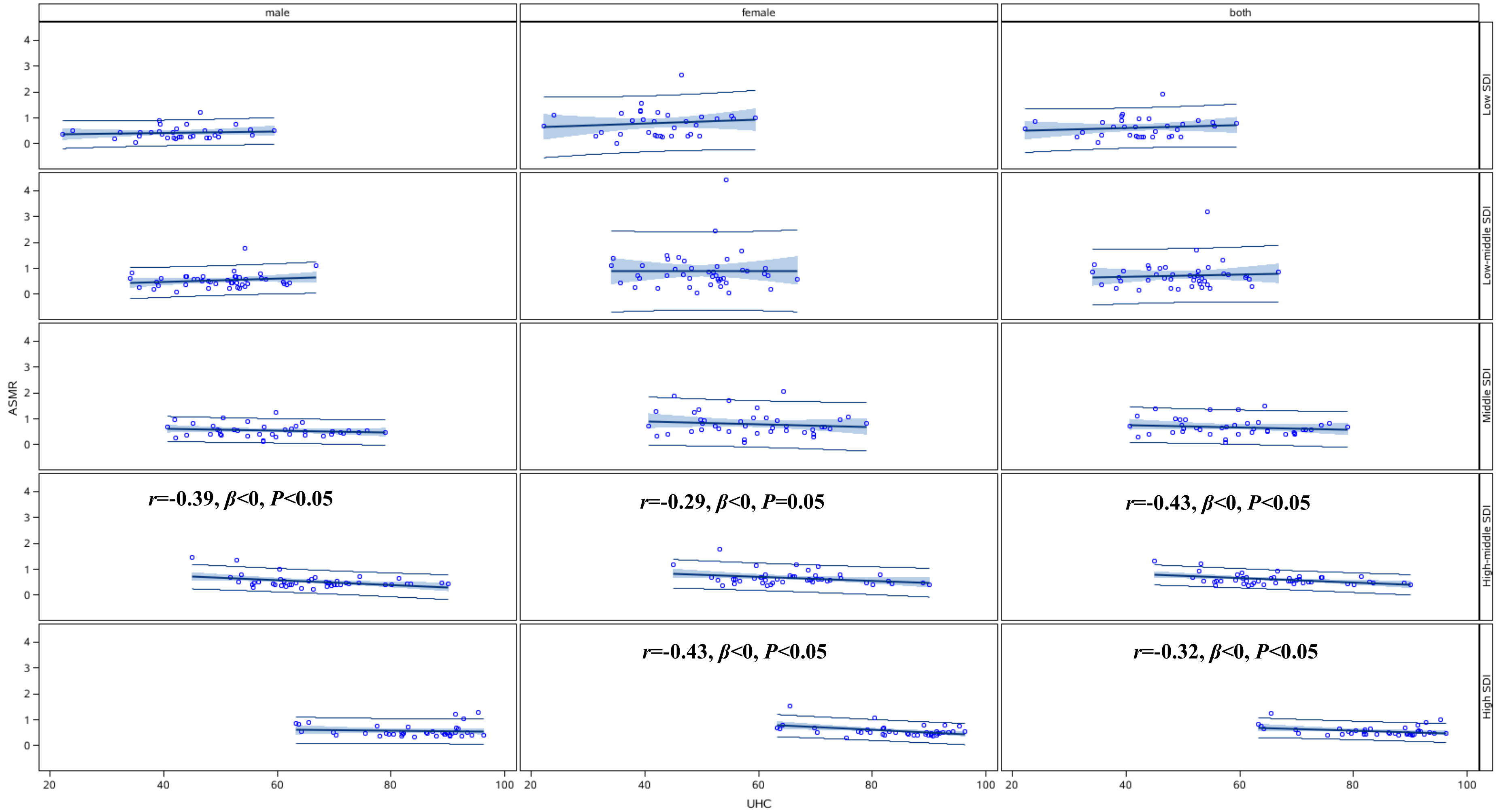

Definition of abbreviations: WB, world bank; ASIR, age-standardized incidence rate; ASMR, age-standardized mortality rate; SDI, sociodemographic index.
